# Profiling and Cellular Analyses of Obesity-Related circRNAs in Neurons and Glia under Obesity-like In Vitro Conditions

**DOI:** 10.3390/ijms24076235

**Published:** 2023-03-25

**Authors:** Danbi Jo, Gwangho Yoon, Yeonghwan Lim, Youngkook Kim, Juhyun Song

**Affiliations:** 1 Department of Anatomy, Chonnam National University Medical School, Hwasun 58128, Republic of Korea; 2 Department of Biochemistry, Chonnam National University Medical School, Hwasun 58128, Republic of Korea

**Keywords:** circular RNAs (circRNAs), obesity, neurons, microglia, astrocytes

## Abstract

Recent evidence indicates that the pathogenesis of neurodegenerative diseases, including Alzheimer’s disease, is associated with metabolic disorders such as diabetes and obesity. Various circular RNAs (circRNAs) have been found in brain tissues and recent studies have suggested that circRNAs are related to neuropathological mechanisms in the brain. However, there is a lack of interest in the involvement of circRNAs in metabolic imbalance-related neuropathological problems until now. Herein we profiled and analyzed diverse circRNAs in mouse brain cell lines (Neuro-2A neurons, BV-2 microglia, and C8-D1a astrocytes) exposed to obesity-related in vitro conditions (high glucose, high insulin, and high levels of tumor necrosis factor-alpha, interleukin 6, palmitic acid, linoleic acid, and cholesterol). We observed that various circRNAs were differentially expressed according to cell types with many of these circRNAs conserved in humans. After suppressing the expression of these circRNAs using siRNAs, we observed that these circRNAs regulate genes related to inflammatory responses, formation of synaptic vesicles, synaptic density, and fatty acid oxidation in neurons; scavenger receptors in microglia; and fatty acid signaling, inflammatory signaling cyto that may play important roles in metabolic disorders associated with neurodegenerative diseases.

## 1. Introduction

Neurodegenerative diseases, including Alzheimer’s disease (AD), have been extensively studied and have a variety of risk factors associated with their initiation and progression [1,2]. For decades, the major hypothesis was that the production and accumulation of amyloid-beta peptides and tau hyperphosphorylation were early factors in AD [3,4]. However, targeting those factors did not completely prevent disease progression. Therefore, several studies have attempted to elucidate the wide range of causes of AD [5]. Recent studies have suggested an association between AD progression and metabolic disorders, such as obesity and type 2 diabetes mellitus (T2DM), based on the common major physiopathology of both diseases, such as insulin resistance and inflammation [6,7,8]. Several studies have shown that the serum from the blood of AD mouse models and AD patients commonly have glucose, insulin, and cholesterol (Chol) imbalances and abnormal secretion of inflammatory cytokines, chemokines, and free fatty acids [9,10,11].

Moreover, it has been reported that patients with obesity and T2DM suffer from impaired memory consolidation and cognitive decline, eventually leading to the development of AD [12,13]. In the central nervous system (CNS), neurons and glia play reciprocal regulatory roles in glucose metabolism, insulin signaling, and lipid metabolism to maintain brain metabolic homeostasis [14]. Neuronal and glial dysfunction damage the maintenance of brain metabolic homeostasis, resulting in cognitive impairment through poor synaptic plasticity [14,15,16]. In the brains of obese patients, pro-inflammatory cytokines, including tumor necrosis factor-alpha (TNF-α) and interleukin-6 (IL-6), induce neuronal apoptosis, abnormal microglial autophagy pathway, and astrocytic mitochondrial dysfunction, resulting in neurite shrinkage and memory impairment [17,18,19]. Moreover, fatty acid imbalances, including elevated saturated palmitic acid (PA), polyunsaturated linoleic acid (LA) deficiency, and elevated low-density lipoprotein cholesterol, cause brain insulin resistance, neuronal apoptosis, microglial inflammatory responses, and impaired astrocytic autophagy, leading to synaptic disruptions and memory loss in the brains of obese patients [20,21,22,23,24]. These significant results warrant further investigation into the key factors related to functional mechanisms in neurons and glia under metabolic imbalances.

Until now, many research groups have focused on the modulation of proteins, mRNAs, DNA epigenetics, and microRNAs in neurons and glia to understand and prevent the development of AD [25,26,27]. However, the functional role of non-coding RNAs (ncRNAs), including circular RNAs (circRNAs), has not been fully elucidated in neurons and glia. CircRNAs are a novel class of regulatory RNAs in which the 3′ end of the downstream exon is covalently bound to the 5′ end of the upstream exon through the back-splicing process [28]. Since most circRNAs are structurally stable, they are likely to play a regulatory role in brain tissue that needs to perform immediate functions; however, in some cases, circRNAs have a fast turnover [29].

Our previous studies have reported the unique expression patterns of specific circRNAs in the brain cortex of obese mice [30], relating to the regulation of the neuronal cell cycle and spatial memory [31]. Similarly, many researchers are trying to elucidate the regulatory functions of circRNAs to understand several diseases, such as cancer and diabetes [32,33,34]. However, studies on the importance of circRNAs in brain metabolic diseases are scarce. CircRNAs are more distributed in the brain than the rest of the body and play a critical role in cellular mechanisms such as synapse formation and neural elongation [29]. Interestingly, a human circRNA, antisense to cerebellar degeneration-related protein 1 (CDR1-AS), is reportedly involved in developmental brain disorders through microRNA (miR)-7 sponging [35]. These studies suggest that elucidating the functions of circRNAs may be an important step in understanding the progression of diseases such as AD.

In this study, we sought to clarify the circRNAs associated with neuronal and glial function under obesity-related in vitro conditions such as neuroinflammation, insulin resistance, and high-saturated fatty acid. Our study provides critical information on various functional roles of candidate circRNAs related to obesity-related neuropathogenesis. Thus, we suggest that these circRNAs may be cardinal regulatory factors in the progression of metabolic-related AD.

## 2. Results

### 2.1. Obesity-Related CircRNAs Were Specifically Expressed in Brain Cells

**Figure 1:**
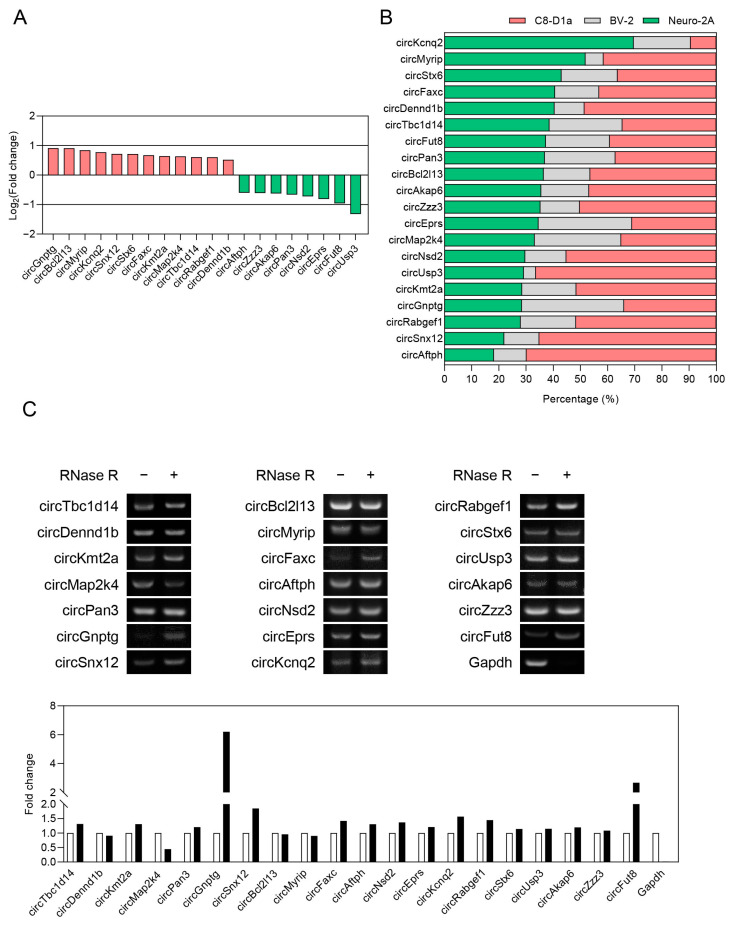
Differential expression and cell-type specificity of obesity-linked circRNAs in the brain. (**A**) A histogram displaying the differential expression of circRNAs in the brain cortices of obese mice compared to wild-type mice. The data are expressed as the average Log_2_ fold change (*n* = 4). (**B**) Stacked bars showing the cell-type specific expression of obesity-linked circRNAs in three mouse-brain cell lines. The total circRNA expression in the three cell lines is set at 100%, and the relative distribution of circRNAs in each cell line is expressed as percentages (*n* = 3). (**C**) Circular structure confirmation of circRNAs. Cropped bands showing the expression of circRNAs and Gapdh in untreated (−) and RNase R-treated (+) Neuro-2A mouse neuroblastoma cells. A histogram displaying changes in expression of circRNAs and Gapdh in RNase R-treated [RNase R (+)] Neuro-2A cells compared to untreated control cells [RNase R (−)]. CircRNAs’ structure confirmation results from a comparative analysis of the expression value of Gapdh mRNA (linear) and the expression value of circRNAs (circular) in the RNase R (+) group. The data are expressed as a relative value of the RNase R-treated group when the untreated control value is 1. Neuro-2A: mouse neuroblastoma cells, BV-2: mouse microglial cells, C8-D1a: mouse astrocytes.

We previously reported a differential expression of circRNAs in the brain cortex of obese mice compared with wild-type mice [30]. Among these circRNAs, we screened 20 that had a log2 fold change greater than 0.5 or lower than −0.5 and with a statistical significance (*p* < 0.05) (Figure 1A). To characterize the distribution of the circRNAs in brain cells, we checked the expression of each circRNA in the mouse cell lines: Neuro-2A neuroblastoma cells, BV-2 microglial cells, and C8-D1a astrocytes (Figure 1B).

We confirmed that the 20 circRNAs were expressed in these cells except for circZbtb16; circKcnq2 was identified as a neuron-dominant circRNA. The identified astrocyte-dominant circRNAs were circZzz3, circNsd2, circUsp3, circKmt2a, circRabgef1, circSnx12, and circAftph. The circRNAs expressed at similar levels in neurons and astrocytes were circMyrip, circStx6, circFaxc, circDennd1b, circAkap6, and circBcl2l13. The circRNAs expressed similarly in the three cell types were circTbc1d14, circFut8, circPan3, circEprs, circMap2k4, and circGnptg. These results show that each obesity-linked circRNA was differentially distributed in brain cells. However, these circRNAs tended to be mainly expressed in neurons and astrocytes, indicating that obesity-linked circRNAs might have regulatory roles in these cells. Through the RNase R treatment and Sanger sequencing analysis, we selected 18 circRNAs with confirmed circular structures and moderate expression levels for further studies (Figure 1C and Figure S1).

### 2.2. Obesity-Related In Vitro Conditions Regulated the Expression of CircRNAs in Brain Cells

To determine how the expressions of the circRNAs were changed by obesity in the brain, we chose seven obesity-related blood serum factors, glucose, insulin, TNF-α, IL-6, PA, LA, and Chol, that mimic obesity in the brain. Various studies have previously studied these factors to examine how obesity impairs brain functions [21]. We introduced each factor to Neuro-2A, BV-2, and C8-D1a cells and established the obesity-like condition models.

**Figure 2:**
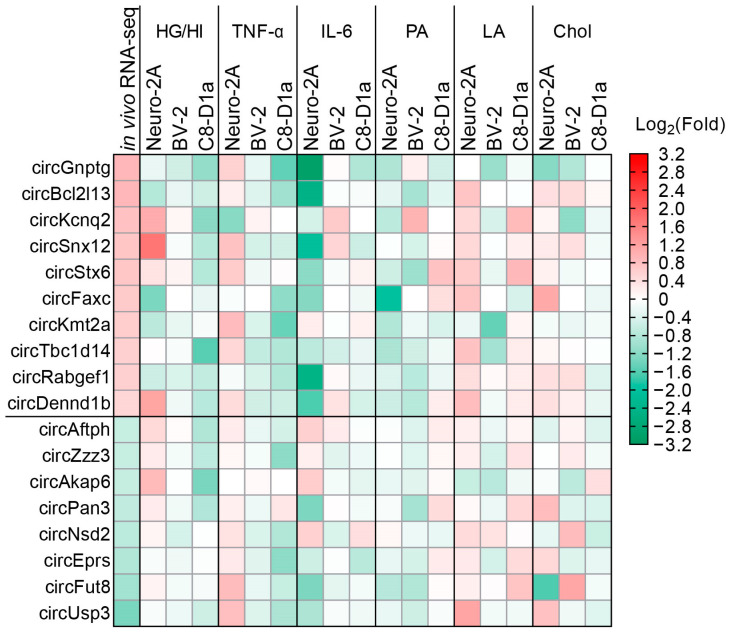
The heatmap displaying the differential expression of obesity-linked circRNAs in three mouse-brain cell lines under obesity-like conditions compared to normal controls. The data for in vivo RNA-seq were obtained and rearranged from our previous report about the transcriptomic analysis of the obese mouse-brain cortex (A reference is referred to in the manuscript). The data are expressed as the average Log_2_ fold change (in vivo RNA-seq: *n* = 4, three cell lines: *n* = 3). The data value and cropped bands corresponding to the heatmap are provided in Supplementary Figures S3–S9. HG/HI: high glucose and insulin concentration, TNF-α: tumor-necrosis factor-alpha, IL-6: interleukin-6, PA: BSA-conjugated palmitic acid, LA: BSA-conjugated linoleic acid, Chol: cholesterol, Neuro-2A: mouse neuroblastoma cells, BV-2: mouse microglial cells, C8-D1a: mouse astrocytes.

We observed that the expressions of various circRNAs were markedly changed in our obesity-like condition models (Figure 2 and Figure S2–S8). We also observed that the expressions of circRNAs matched the unique expression pattern of circRNAs in the obesity-like condition models (Figure 2) [30]. High glucose and insulin concentrations (HG/HI) significantly regulated the expression of circSnx12 in neurons, circUsp3 in microglia, and circSnx12 in astrocytes (Figure 2 and Figure S3). TNF-α markedly regulated the expression of circGnptg in neurons and circKmt2a, circGnptg, circFaxc, and circZzz3 in astrocytes (Figure 2 and Figure S4), indicating that TNF-α has a significant effect on circRNA expression in astrocytes. Furthermore, we observed that IL-6 significantly regulated circTbc1d14 expression in microglia only, suggesting that IL-6 plays a minimal role in regulating the expression of circRNAs in this assay (Figure 2 and Figure S5). BSA-conjugated PA significantly regulated the expression of circKcnq2 in neurons and circGnptg in astrocytes (Figure 2 and Figure S6). BSA-conjugated LA significantly regulated the expression of circDennd1b, circRabgef1, circFaxc, and circUsp3 in neurons; circGnptg, circEprs, and circStx6 in microglia; and circStx6 in astrocytes (Figure 2 and Figure S7), indicating that contrary to PA, LA has a significant effect on circRNA expression in neurons. Chol significantly regulated the expression of circDennd1b, circPan3, circEprs, circFaxc, and circFut8 in neurons; circPan3, circGnptg, and circKcnq2 in microglia; and circAftph and circUsp3 in astrocytes (Figure 2 and Figure S8), indicating that Chol has a significant effect on circRNA expression in brain cells.

We further confirmed that the obesity-like conditions did not significantly affect the expression of most host genes that corresponded to each of their circRNAs (Supplementary Figure S9), indicating that obesity-like conditions regulate circRNA expression without affecting the transcription of host genes. These results comprehensively suggest that the expressions of circRNAs are significantly changed in our obesity-like condition models. These results also indicate that the unique expression patterns of circRNAs in the brains of obese mice were because of the obesity-related serum factors used in our obesity-like condition models. Moreover, these results ascertain that our obesity-like condition models are proper for the functional analysis of obesity-linked circRNAs.

### 2.3. Obesity-Related In Vitro Conditions Affected the Cell Type-Specific Functions of Brain Cells

**Figure 3:**
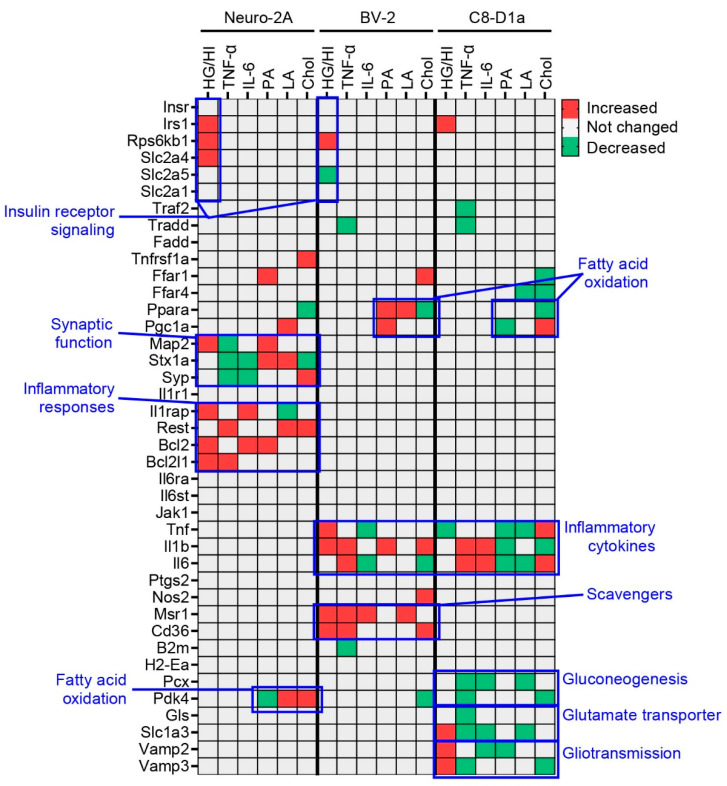
The heatmap displays upregulated and downregulated genes associated with functions by cell type and obesity-related serum factor in three mouse-brain cell lines under obesity-like conditions compared to normal controls. The blue letters and boxes represent clusters of genes that function similarly. The data are expressed as “increased” when the gene expression was significantly increased in cells under obesity-like conditions compared to controls (Red). “Not changed” indicates no significant changes in gene expression in cells under obesity-like conditions compared to normal controls (Silver). “Decreased” indicates the gene expression was significantly decreased in cells under obesity-like conditions compared to normal controls (Green). The data values used in the heatmap are expressed as a histogram in Supplementary Figure S11A–F. HG/HI: high glucose and insulin concentration, TNF-α: tumor-necrosis factor-alpha, IL-6: interleukin-6, PA: BSA-conjugated palmitic acid, LA: BSA-conjugated linoleic acid, Chol: cholesterol, Neuro-2A: mouse neuroblastoma cells, BV-2: mouse microglial cells, C8-D1a: mouse astrocytes.

**Figure 4:**
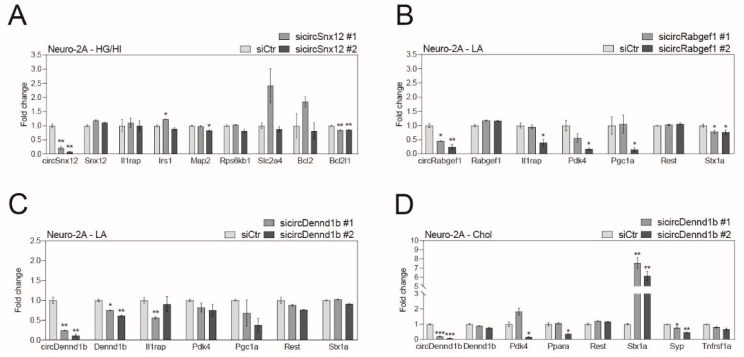
Histograms displaying the differential expression of functional genes after depletion of circRNAs in neuronal cell lines under obesity-like conditions. (**A**) Expression changes of genes associated with neuronal responses by high glucose and insulin concentration (HG/HI) after circSnx12 depletion in mouse neuroblastoma cells (Neuro-2A). (**B**) Expression changes of genes associated with neuronal responses by BSA-conjugated linoleic acid (LA) after circRabgef1 depletion in Neuro-2A cells. (**C**) Expression changes of genes associated with neuronal responses by LA after circDennd1b depletion in Neuro-2A cells. (**D**) Expression changes of genes associated with neuronal responses by cholesterol (Chol) after circDennd1b depletion in Neuro-2A cells. In (**A**–**D**), the data are presented as the mean ± standard error of the mean (SEM) (*n* = 3), and statistical significance was determined using an unpaired two-tailed *t*-test with Welch’s correction; * *p* < 0.05, ** *p* < 0.01, *** *p* < 0.001.

**Figure 5:**
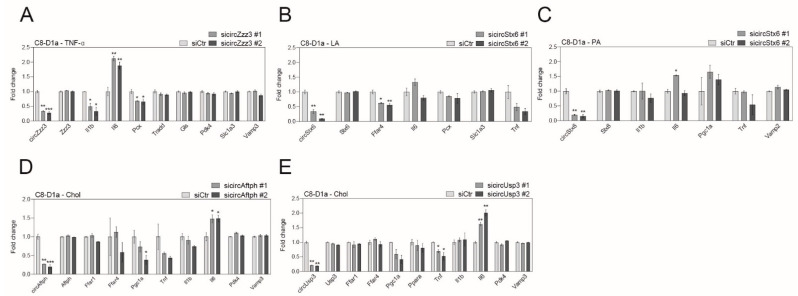
Histograms displaying the differential expression of functional genes after depletion of circRNAs in an astrocyte cell line under obesity-like conditions. (**A**) Expression changes of genes associated with astrocytic responses by TNF-α after circZzz3 depletion in C8-D1a cells. (**B**) Expression changes of genes associated with astrocytic responses by LA after circStx6 depletion in C8-D1a cells. (**C**) Expression changes of genes associated with astrocytic responses by PA after circStx6 depletion in C8-D1a cells. (**D**) Expression changes of genes associated with astrocytic responses by Chol after circAftph depletion in C8-D1a cells. (**E**) Expression changes of genes associated with astrocytic responses by Chol after circUsp3 depletion in C8-D1a cells. The cropped band used for analysis is in Supplementary Figure S13. In (Figure S13A–I), the data are presented as the mean ± standard error of the mean (SEM) (*n* = 3), and statistical significance was determined using an unpaired two-tailed *t*-test with Welch’s correction; * *p* < 0.05, ** *p* < 0.01, *** *p* < 0.001.

We then used a semi-quantitative PCR analysis to examine the change in the expression of marker genes in our obesity-like condition models. First, we identified gene sets related to the function of each obesity-related serum factor: HG/HI (*Insr*, *Irs1*, *Rps6kb1*, *Slc2a4*, *Slc2a5*, *Slc2a1*), TNF-α (*Tnfrsf1a*, *Tradd*, *Traf2*, *Fadd*), IL-6 (*Il6ra*, *Il6st*, *Jak1*), and fatty acids (PA, LA, Chol; *Ffar1*, *Ffar4*, *Ppara*, *Pdk4*, *Pgc1a*). We also identified gene sets related to the function of each brain cell type: neurons (*Map2*, *Stx1a*, *Syp*, *Il1ra1*, *Il1rap*, *Rest*, *Bcl2*, *Bcl2l1*, *Il6ra*, *Tnfrsf1a*), microglia (*Tnf*, *Il1b*, *Il6*, *Ptgs2*, *Nos2*, *Msr1*, *Cd36*, *B2m*, *H2-Ea*), and astrocytes (*Pcx*, *Pdk4*, *Gls*, *Slc1a3*, *Vamp2*, *Vamp3*, *Tnf*, *Il1b*, *Il6*). We then listed a cluster of unique genes that were significantly upregulated or downregulated in our obesity-like condition models (Figure 3). We observed that the obesity-like conditions regulated the expression of genes associated with insulin receptor signaling (*Irs1*, *Rps6kb1*, *Slc2a4*), synaptic function (*Map2*, *Stx1a*, *Syp*), inflammatory responses (*Il1rap*, *Rest*, *Bcl2*, *Bcl2l1*), and fatty acid oxidation (*Pdk4*) in neurons; insulin receptor signaling (*Rps6kb1*, *Slc2a5*), fatty acid oxidation (*Ppara*, *Pgc1a*), inflammatory cytokines (*Tnf*, *Il1b*, *Il6*), and scavenger receptors (*Msr1*, *Cd36*) in microglia; and fatty acid oxidation (*Ppara*, *Pgc1a*), inflammatory cytokines (*Tnf*, *Il1b*, *Il6*), gluconeogenesis (*Pcx*, *Pdk4*), glutamate transporters (*Gls*, *Slc1a3*), and gliotransmission (*Vamp2*, *Vamp3*) in astrocytes (Figure 4, Figure 5 and Figure S10). These comprehensive results indicate that the obesity-related serum factors affected the overall function of the brain by regulating the functional genes of neurons, microglia, and astrocytes. Moreover, it suggests that obesity-linked circRNAs might control brain functions by regulating altered genes.

### 2.4. Obesity-Related CircRNAs Work as Important Factors for Brain Cell Function

To examine the role of obesity-linked circRNAs in brain cells, we first assessed circRNAs that are well conserved in humans by analyzing the publicly available Gene Expression Omnibus (GEO) dataset and the expression measurement from human SH-SY5Y neuroblastoma cells. We then sorted circRNAs showing unique expression patterns in both obesity-like condition models and obese mouse brains (Figure 2). We found that five circRNAs (circStx6, circDennd1b, circUsp3, circAftph, circZzz3) are well conserved in the human brain cortex (Supplementary Figure S11). Furthermore, we confirmed two additional circRNAs (circSnx12 and circRabgef1) that were not listed in the GEO dataset but stably expressed in human SH-SY5Y cells (Supplementary Figure S11). Thus, we selected these eleven circRNAs for functional analyses in our obesity-like condition models.

We designed two independent siRNAs that bind to the back-splicing junction of each circRNA for the specific depletion of circRNAs without affecting their host mRNA (Supplementary Figure S12). We confirmed that the siRNAs sufficiently depleted the expression of each obesity-linked circRNA (Figure 4 and Figure 5; 60–90% expression depletion). We then observed that obesity-linked circRNAs regulated various functions in brain cells under obesity-like conditions (Figure 4 and Figure 5). CircSnx12 depletion significantly decreased the expression of *Bcl2l1* in HG/HI-induced neurons, indicating that circSnx12 has a role in the inflammatory response pathway in this model (Figure 4A and Figure S13A). Downregulation of circRabgef1 significantly decreased the expression of *Stx1a* in LA-treated neurons, suggesting that circRabgef1 is involved in the cascade of synaptic vesicles and synaptic density in this model (Figure 4B and Figure S13B). CircDennd1b depletion significantly decreased the expression of its host gene in LA-treated neurons without affecting functional genes (Figure 4C and Figure S13C). However, circDennd1b knockdown significantly decreased the expression of *Stx1a* and *Syp* in Chol-treated neurons without affecting its host gene expression (Figure 4D and Figure S13D), indicating that circDennd1b has a distinct role depending on obesity-related serum factors and is involved in the formation of synaptic vesicles and density in Chol-treated neurons. CircZzz3 downregulation significantly regulated the expression of *Il1b*, *Il6*, and *Pcx* in TNF-α-treated astrocytes, indicating that circZzz3 is involved in inflammatory cytokine production and gluconeogenesis in this model (Figure 5A and Figure S13E). CircStx6 downregulation significantly regulated the expression of *Ffar4* in LA-treated astrocytes, indicating that circStx6 is involved in fatty acid receptor signaling in this model (Figure 5B and Figure S13F). However, circStx6 depletion did not show gene expression changes in PA-treated astrocytes (Figure 5C and Figure S13G), indicating that there might be other roles we did not examine in this assay and that circStx6 may also have distinct roles depending on obesity-like conditions. The downregulation of circAftph significantly increased the expression of *Il6* in Chol-treated astrocytes, indicating that these circRNAs involve inflammatory cytokine production in this model (Figure 5D and Figure S13H). CircUsp3 knockdown significantly increased the expression of *Msr1* and *Cd36* in HG/HI-induced microglia, indicating that circUsp3 plays a role in scavenger-receptor signaling in this model (Figure 5E and Figure S13I). CircUsp3 depletion significantly regulated the expression of *Tnf* and *Il6* in Chol-treated astrocytes, indicating that circUsp3 is involved in inflammatory cytokine secretion in this model (Figure 5E and Figure S13I).

Interestingly, we frequently observed that many circRNAs were involved in regulating *Il6* expression in astrocytes exposed to TNF-α and Chol, indicating that obesity-linked circRNAs may be key factors that regulate the IL-6 cytokine production in astrocytes. Together with the results that our selected circRNAs might involve the neuronal inflammatory response, neuronal synapse formation, neuronal fatty acid oxidation, and microglial scavengers, our results suggest that obesity-linked circRNAs have pivotal and multifunctional roles in the obese brain. Our obesity-linked circRNA profiling in brain cells might help to unveil obesity-related brain dysfunction and neurodegenerative diseases.

### 2.5. Analysis of the Protein Interaction for Obesity-Related CircRNAs

**Figure 6:**
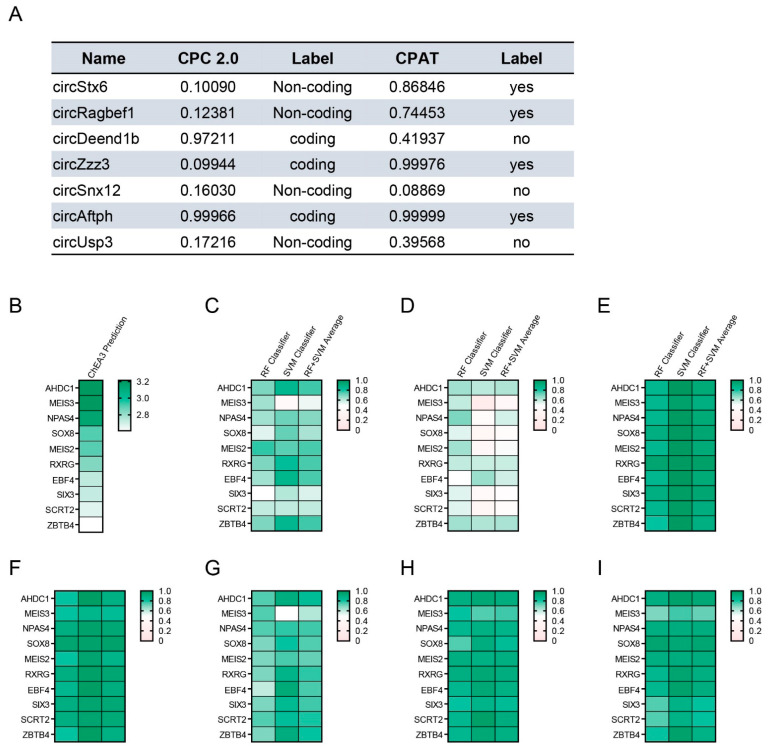
Characterization of circRNAs (**A**) The protein-coding potential of circRNAs was analyzed by CPC 2.0 (expressed as coding or non-coding) and CPAT (expressed as yes or no) tools. (**B**) The prediction of protein factors using ChEA3 that may regulate transcription of genes affected by HFD in the RNA-seq data. The color bar expresses the top ten ranking of the integrated score for ChEA3. (**C**) The interaction probability between circStx6 and transcription factors was predicted using RPIseq. (**D**) The interaction probability between circRagbef1 and transcription factors was predicted using RPIseq. (**E**) The interaction probability between circDeen1b and transcription factors was predicted using RPIseq. (**F**) The interaction probability between circZzz3 and transcription factors was predicted using RPIseq. (**G**) The interaction probability between circSnx12 and transcription factors was predicted using RPIseq. (**H**) The interaction probability between circAftph and transcription factors was predicted using RPIseq. (**I**) The interaction probability between circUsp3 and transcription factors was predicted using RPIseq. In (**C**–**I**), the color bar indicates the interaction probability score (0–1) between circRNAs and transcription factors. RF: random forest, SVM: support vector forest.

Some circRNAs have been reported to encode proteins and peptides [36,37]. We analyzed the protein-coding potential of candidate circRNAs to confirm the possibility of protein translation (Figure 6A). The protein-coding potential of seven circRNAs was confirmed using two prediction tools: CPC 2.0 and CPAT [38,39]. It was confirmed that some circRNAs, except circSnx12 and circUsp3, are highly likely to code proteins. To identify the transcriptional regulatory mechanism of circRNA candidates, we first identified transcription factors that regulate differentially expressed genes in previously reported RNA-seq data [30]. Using the prediction tool ChEA3, we selected the top ten transcription factors involved in the transcriptional control of 459 genes whose *p*-values were ≤ 0.05 in the Cuffnorm results (Figure 6B) [40]. We confirmed the interaction between each circRNA and transcription factor using the RNA-protein interaction prediction tool RPIseq (Figure 6C–I) [41]. Among various factors, neuronal PAS domain protein 4 (NPAS4), SRY-box transcription factor 8 (SOX8), and retinoid X receptor gamma (RXRG) were shown to be highly likely to interact with circSnx12 and circUsp3, which have low protein-coding potential (Figure 6G,I). NPAS4 is known to play an important role in contextual memory formation in the CA3 region of the hippocampus as a transcriptional factor [42]. SOX8 has been reported to enhance the astrogenesis of neural stem and precursor cells by targeting Nfia [43]. RXRG deficiency is known to impair spatial memory in mice and reduce mGluR-mediated synaptic plasticity [44,45]. Based on these results, the cellular mechanisms behind the interactions between obesity-related circRNAs and transcriptional factors in neurons and glia in the condition of obesity may be elucidated.

## 3. Discussion

Herein we comprehensively analyzed specifically or similarly expressed circRNAs in brain cells, such as neurons, microglia, and astrocytes, exposed to diverse obesity-related in vitro conditions. This study is based on our previous report of distinct expression patterns of circRNAs in the brain cortices of obese mice [30]. We profiled significant obesity-related circRNAs that may have regulatory roles in neuropathological mechanisms in neurodegenerative diseases such as AD. From our analysis, we speculate that obesity-related circRNAs are mainly distributed in neurons and astrocytes, suggesting that circRNAs distributed in these cells play regulatory roles in obesity-related brain dysfunction. Moreover, we profiled unique obesity-related circRNAs in our obesity-like in vitro models. Among these circRNAs, we selected those expressed in patterns similar to those of the transcriptomic analysis data from obese mouse brains for further functional analysis [30]. We consider that each cell type in the brain has different intrinsic functions and that circRNAs are likely to have different cell-specific expressions and functions [46].

We selected 11 human-conserved circRNAs that may have pivotal roles in obesity-induced brain dysfunction. Among these circRNAs, the functions in human diseases of four, including circRabgef1, circUsp3, circZzz3, and circAftph, have not yet been identified. Moreover, for circRNAs whose roles have been previously reported, including circSnx12, circDennd1b, and circStx6, their specific functions have not yet been completely understood in the brain. For example, circDennd1b is involved in atherosclerosis by regulating Chol efflux through a miRNA-17-5p sponge [47], but its role in the brain is largely unknown.

We established obesity-like condition models using brain cell lines to mimic the environment of the brains of obese individuals. These models were based on reports on the characteristics of blood serum profiling in high-fat-fed mice, rats, monkeys, and humans with obesity models [48,49,50,51,52,53]. Blood serum factors in obesity are important mediators in the onset of neurodegenerative diseases since the blood-brain barrier (BBB) is disrupted or loosened in obesity conditions, and consequently, blood serum factors can be delivered into brain tissue resulting in memory loss [54,55,56]. Other studies also showed that the blood serum in obese contains higher pro-inflammatory cytokine levels, such as TNF-α and IL-6, leading to neuroinflammation and spatial memory loss [57,58]. Moreover, a high Chol level in obesity damages the metabolic crosstalk between neurons and glia and aggravates synaptic formation [59,60]. Furthermore, an increased level of PA, a saturated fatty acid, impairs autophagy and insulin signaling in neurons in obesity [61]. An elevated PA level accelerates apoptosis and inflammatory responses in obesity by activating glia [62]. Furthermore, the high glucose level in obesity is accompanied by insulin resistance and, subsequently, increased neuronal cell loss and BBB permeability, leading to memory loss [63,64]. Another study showed that excessive linoleic polyunsaturated fatty acid is related to hypothalamic inflammation, thus boosting weight gain [65] and affecting insulin resistance [66]. In obesity, fatty acid composition is an important factor for predicting the progression of lipid dysregulation and inflammation in obesity. Some studies presented that these characteristics of blood serum in obesity are considerably associated with neurodegenerative diseases such as AD [67,68,69,70]. Thus, we selected obesity-related in vitro conditions, including glucose, insulin, TNF-α, IL-6, PA, LA, and Chol, to mimic obesity status.

In the present study, each obesity-related in vitro condition led to alterations in genes related to synaptic function, inflammatory responses, insulin receptor signaling, fatty acid oxidation, scavenger receptor signaling, gluconeogenesis, gliotransmission, and glutamate transporters in neurons and glia. These obesity-related in vitro condition-induced expression changes indicate that metabolic imbalances influence insulin receptor signaling, synaptic function, and inflammatory responses in neurons and inflammatory cytokine secretion, scavenger ability, and glutamate transporters in glia. Several studies mentioned that chronic metabolic disorders, such as obesity and T2DM, modulate the inflammatory response, synaptic dysfunction, fatty acid oxidation, and microglial scavenger function in the brain [71,72]. Obesity impairs brain function and synapse formation by impairing insulin signaling [73]. In neurons, insulin receptor signaling-related genes, such as *Irs1* and *Slc2a4*, could affect the secretion of excitatory glutamate neurotransmitters [74], neuroinflammation [75], and memory formation [76]. Moreover, in neurons, synaptic formation-related genes, such as *Map2* and *Syp*, are related to memory consolidation and synaptic plasticity [77].

Inflammatory-related genes, such as *Bcl2*, are linked to neuronal Ca^2+^ signaling [78], neurogenesis, and apoptosis [79]. A recent study suggested that obesity triggers neuroinflammation and fatty acid oxidation in the brain [61]. In microglia, scavenger receptor-related genes, such as macrophage scavenger receptor 1 (*Msr1*) and *CD36,* are related to low-density Chol uptake [72], amyloid beta phagocytosis [80], and lipid metabolism homeostasis [81]. In microglia and astrocytes, fatty acid oxidation-related genes, such as *Ppara* and *Pgc1a*, are associated with autophagy, neuronal proliferation, memory formation [82,83], microglia polarization [84], and apoptosis [85]. In astrocytes, glutamate transporter-related genes, such as *Gls*, are related to excitatory neurotransmitter secretion and synapse transmission [86]. A recent study mentioned that obesity is involved in neuroplasticity and cognitive decline through astroglial dysfunction, such as gluconeogenesis, glutamate signaling, and gliotransmission [87].

Therefore, we assume that our obesity-like in vitro conditions triggered neuronal dysfunction related to insulin signaling and synaptic formation and glial dysfunction related to phagocytosis and Chol uptake, leading to cognitive impairment.

In Figure 4 and Figure 5, we observed changes in the expression of several genes in neurons and glial cells after silencing specific circRNAs using siRNAs. In our data, circDennd1b depletion using siRNA significantly regulated genes related to synaptic vesicles and synaptic formation, such as *Stx1a* and *Syp*, in neurons [88] under Chol exposure. This result suggests that the role of circDennd1b may be related to synaptic plasticity in neurons under Chol-enriched obesity conditions. In particular, we found a significantly abnormal expression of IL-6 in astrocytes after obesity-related circRNA depletion under obesity-related in vitro conditions. Considering that astrocyte-specific IL-6 knockout mice showed increased body weight, and cerebral IL-6 overexpressing mice resisted diet-induced obesity, IL-6 might be a key cytokine associated with astrocyte function by regulating several circRNAs in obesity [89,90]. This result suggests that the modulation of obesity-related circRNAs might affect the expression of IL-6 in astrocytes to regulate neuronal and glial function in the brain of obese individuals.

Even though the effects of our candidate circRNAs on brain function have not been studied until now, their functions in metabolic disorders in neuronal and glial cells have yet to be identified.

In this study, we investigated circRNAs expressed in CNS cells exposed to an obesity-like environment. We profiled candidate circRNAs that show expression changes in the brain cortex of obese mice. In addition, we selected circRNAs that show expression changes under obesity similar to in vitro conditions and are expressed in patterns similar to transcriptomic data from obese mouse brains. Judging from our findings in this study, we hypothesize that each circRNA expressed in CNS cells exhibits cell-specific expression changes and contributes to brain function in the obese brain.

Since a large part of our data was produced based on immortalized or tumoral cell lines derived from the brain cells, it may less reflect the physiology of the actual nervous system. Therefore, further studies are necessary to verify the expression and functions of each circRNA in in vitro obesity cells obtained by isolating primary cortical and hippocampal neurons, microglia and astrocytes from mice. Moreover, an in vivo study is necessary to determine whether the regulation of circRNAs expression in the mouse brain affects cognitive function in animal models.

Even though there are several limitations, our data show the potential of candidate cirRNAs related to neuropathological issues in the brain with obesity. Thus, we suggest that functional studies on circRNAs in CNS cells of obese brain are necessary for an appropriate therapeutic approach to the neuropathological problems of obesity brain.

## 4. Materials and Methods

### 4.1. Drug Treatment

D-glucose (Gibco, Waltham, MA, USA), insulin (Sigma Aldrich, Saint Louis, MI, USA), recombinant TNF-α (Abcam, Cambridge, UK), recombinant IL-6 (Abcam, Cambridge, UK), PA (Sigma Aldrich, Saint Louis, MI, USA), LA (Sigma Aldrich, Saint Louis, MI, USA), and Chol (Sigma Aldrich, Saint Louis, MI, USA) were used to establish the obesity-like models. D-glucose, TNF-α, and IL-6 were diluted using sterilized 1X phosphate-buffered saline (PBS). Insulin was diluted using sterilized acidic distilled water, and the pH was adjusted to be between 2.0 and 3.0 with diluted HCl. Chol was diluted using absolute ethanol (Thermo Fisher Scientific, Waltham, MA, USA). PA and LA were conjugated with bovine serum albumin (BSA; GenDEPOT, Barker, TX, USA). For BSA conjugation, PA and LA were diluted using absolute ethanol, then boiled at 40 °C for at least two hours while vortexing. The solution was filtrated using a syringe filter (0.2 μm; Millipore, Saint Louis, MI, USA) and mixed with a 10% BSA solution at a 1:100 ratio. The cells were treated with D-glucose (4.5 g/L [91]), insulin (100 nM [91]), TNF-α (25 ng/mL [92]), IL-6 (25 ng/mL [93]), BSA-conjugated PA (50 μM [94]), BSA-conjugated LA (50 μM [95]), and Chol (50 μM [96]) for 48 h.

### 4.2. Cell line and Culture Conditions

Mouse Neuro-2A neuroblastoma cells, mouse BV-2 microglial cells, mouse C8-D1a astrocytes, and human SH-SY5Y neuroblastoma cells were purchased from the American Type Culture Collection (ATCC, Manassas, VA, USA). Neuro-2A and SH-SY5Y cells were cultured in Dulbecco′s Modified Eagle′s Medium (DMEM, WELGENE, Gyeongsan Republic of Korea) supplemented with 10% fetal bovine serum (FBS, Millipore, USA), 1 mM sodium pyruvate (Thermo Fisher Scientific, Waltham, MA, USA), and 100 U/mL penicillin–streptomycin (Thermo Fisher Scientific, Waltham, MA, USA). BV-2 cells were cultured in DMEM containing 5% FBS and 100 U/mL penicillin–streptomycin. C8-D1a cells were cultured in DMEM supplemented with 10% FBS and 100 U/mL penicillin–streptomycin. Cells were cultured at 37 °C in the presence of 5% CO_2_. The medium was replaced once every two days. The cells were subcultured into multi-well plates using prewarmed 1X PBS (GENEALL, Seoul, Republic of Korea) and 0.25% trypsin (Thermo Fisher Scientific, Waltham, MA, USA).

### 4.3. siRNA Design and Transfection

The siRNAs to suppress circRNA expression were designed as previously reported [97]. siDESIGN Center (https://horizondiscovery.com/en/products/tools/siDESIGN-Center/ accessed on 4 May 2022) and i-Score Designer (https://www.med.nagoya-u.ac.jp/neurogenetics/i_Score/i_score.html/ accessed on 4 May 2022) were used to identify the siRNAs that targeted the back-splicing junction of circRNAs. These siRNAs and the AccuTarget negative control siRNA were synthesized by Bioneer (Republic of Korea). The sequences of the siRNAs used are listed in Supplementary Table S1.

The siRNAs were transfected using Lipofectamine 3000 (Thermo Fisher Scientific, Waltham, MA, USA) according to the manufacturer′s instructions. siRNAs with a final concentration of 30 nM were transfected into brain cells. The transfected cells were incubated for six hours. The medium was replaced with a growth medium containing the reagent for obesity-like conditions and incubated for 48 h.

### 4.4. RNA Isolation and Semi-Quantitative Polymerase Chain Reaction

RNA was extracted using TRIzol reagent (Thermo Fisher Scientific, Waltham, MA, USA) according to the manufacturer’s instructions. The RNA quantification was performed using a NanoPhotometer (IMPLEN, München, Germany), and reverse transcription of RNA to complementary DNA (cDNA) was performed using random hexamers and RevertAid reverse transcriptase (Thermo Fisher Scientific, Waltham, MA, USA). A semi-quantitative polymerase chain reaction (PCR) was conducted using nTaq DNA polymerase (Enzynomics, Daejeon, Republic of Korea) in Master cycler Nexus X2 (Eppendorf, Hamburg, Germany). The results of the semi-quantitative PCR were evaluated by electrophoresis using a 2% agarose gel. The gel was analyzed using Image J (V1.53c) provided by the National Institutes of Health (NIH) [98]. The expression of circRNAs and mRNA was normalized against the expression of Gapdh. The primer sequences are listed in Supplementary Table S1.

### 4.5. Confirmation of the Circular Structure of the CircRNAs

To verify the circular structure of the circRNAs, total RNA was treated with RNase R (Biosearch Technologies, Hoddesdon, UK), which only degrades linear RNAs. The total RNA and RNase R mixture was incubated at 37 °C for 5 min. Then RNase R was inactivated by incubating the mixture at 95 °C for 3 min. The RNA was reverse-transcribed into cDNA using random hexamers and RevertAid reverse transcriptase. Finally, a semi-quantitative PCR was performed to amplify the circRNAs of interest. The PCR product was electrophoresed on a 2% agarose gel. The sequences of the PCR product were verified using Sanger sequencing (Solgent, Daejeon, Republic of Korea).

### 4.6. Analysis of CircRNA Function

We used the coding potential calculator 2 (CPC 2.0, http://cpc2.gao-lab.org/ accessed on 25 January 2023) and the coding potential assessment tool (CPAT, http://lilab.research.bcm.edu/ accessed on 25 January 2023) for the prediction of coding potential for the selected circRNAs [38,39]. To predict circRNA-interacting proteins, we performed the analysis as previously described [31]. Using the ChEA3 tools [40], we selected the top ten transcription factors that regulate the 459 differentially expressed genes with *p*-values less than 0.05 analyzed from the cortex of mice fed with a high-fat diet [30]. For each of these transcription factors, we predicted the probability of their interactions with each circRNA using the RPIseq tool [41].

### 4.7. Statistical Analyses

Data are represented as the mean ± standard error of the mean (SEM). The group sample size was typically set to three for our experiments to optimize the efficiency and power of the statistical tests. The normal distribution and similar variance within each comparison group of data were checked before the statistical tests. An unpaired two-tailed *t*-test with Welch’s correction was used to analyze the comparisons between the control and experimental samples. Statistical significance was established when the *p*-value was less than 0.5.

## Data Availability

The data presented in this study are available within the article. Other data related to this study are available on request from the corresponding author.

## Supplementary Materials

The following supporting information can be downloaded at: https://www.mdpi.com/article/10.3390/ijms24076235/s1.
