# Supplementary material for: Profiling and Cellular Analyses of Obesity-Related circRNAs in Neurons and Glia under Obesity-like In Vitro Conditions
Source: Int J Mol Sci. 2023 Mar 25;24(7):6235. doi: 10.3390/ijms24076235 (PMC10094513; doi:10.3390/ijms24076235)
Supplement: Supplementary file 1 [file ijms-24-06235-s001.zip › supplementary files/Supplementary Figure Legends-revised.pdf]

Supplementary Figure Legends

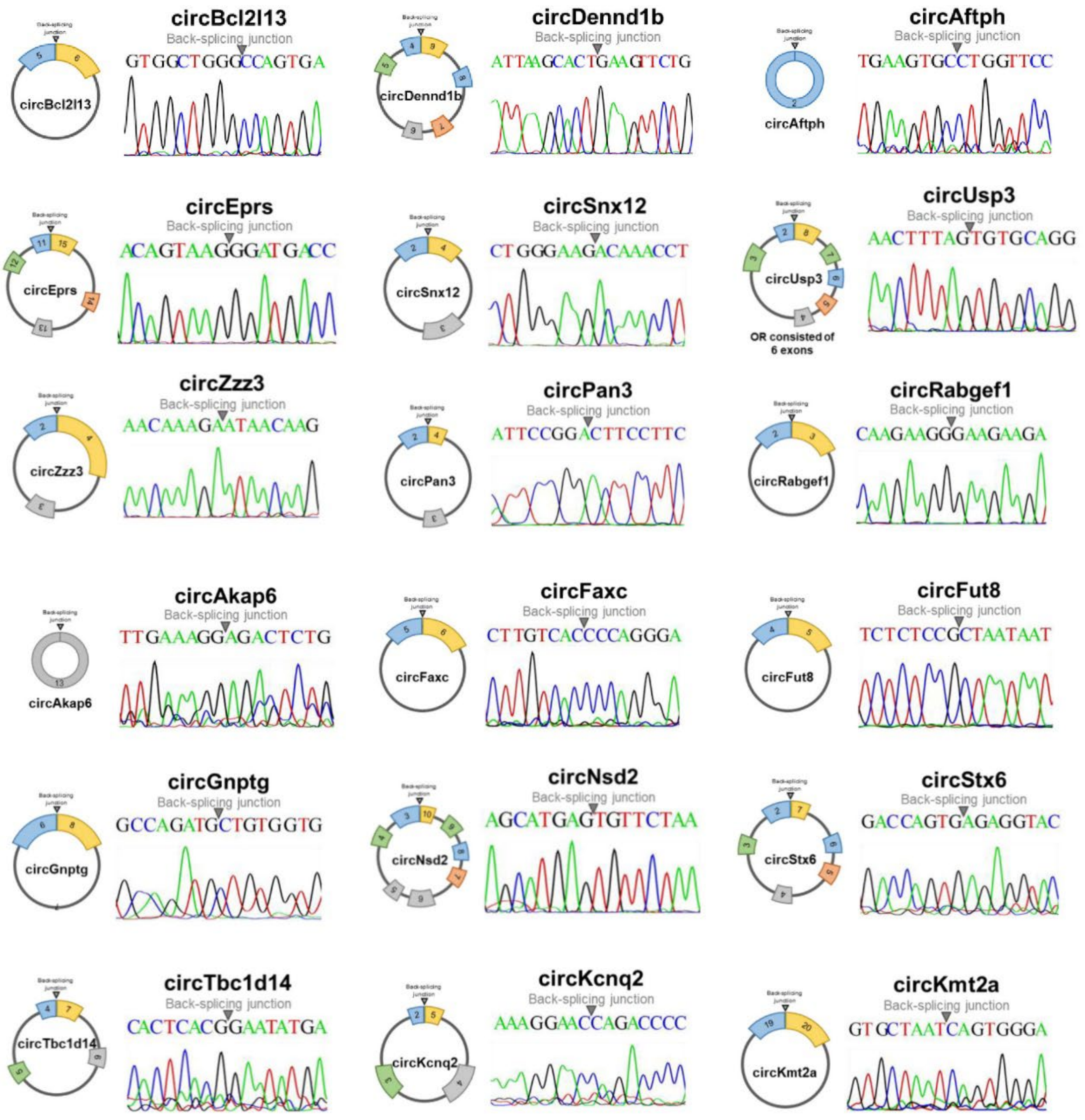

Supplementary Figure S1. Illustration showing the structure and back-splicing junction sequence of circRNAs for circular structure confirmation. The solid circular line and blocked circular arc in each circRNA model indicate the introns and exons, respectively. The number in the exon arc represents the exon number designated in the Genome browser database. The silver triangle in each circRNA model and sequence indicates the back-splicing junction site.

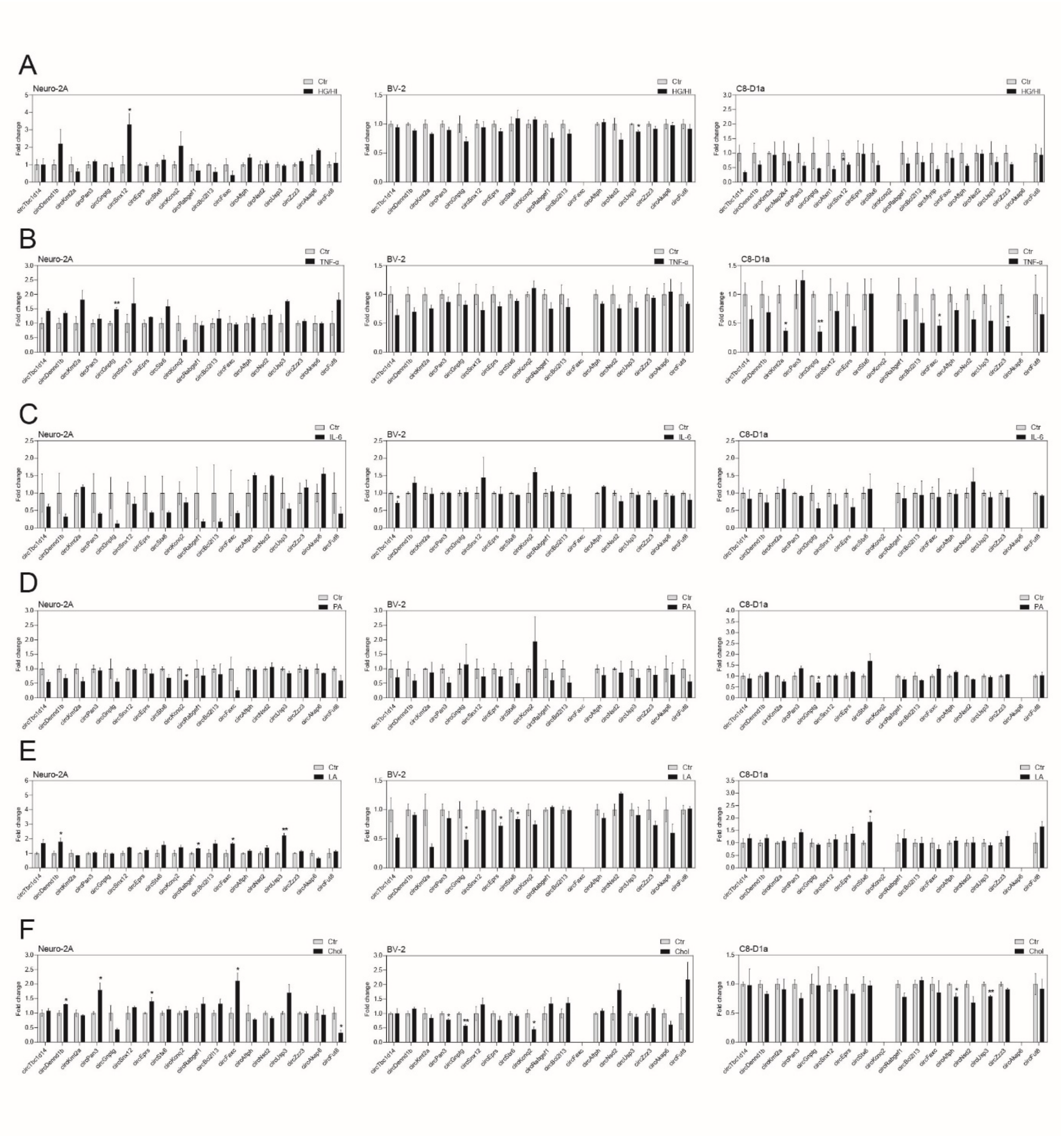

Supplementary Figure S2. Differential expression of obesity-linked circRNAs in three mouse-brain cell lines under obesity-like conditions. **A.** Histograms show altered expression of circRNAs by high glucose and insulin concentration (HG/HI) in mouse neuroblastoma (Neuro-2A), mouse microglial (BV-2), and mouse astrocytes (C8-D1a) compared to each untreated control (Ctr). **B.** Histograms show an altered expression of circRNAs by tumor necrosis factor-alpha (TNF-α) in Neuro-2A, BV-2, and C8-D1a cells compared to each Ctr. **C.** Histograms show an altered expression of circRNAs by interleukin-6 (IL-6) in Neuro-2A, BV-

2, and C8-D1a cells compared to each Ctr. **D.** Histograms show an altered expression of circRNAs by BSA-conjugated palmitic acid (PA) in Neuro-2A, BV-2, and C8-D1a cells compared to each Ctr. **E.** Histograms show an altered expression of circRNAs by BSA-conjugated linoleic acid (LA) in Neuro-2A, BV-2, and C8-D1a cells compared to each Ctr. **F.** Histograms show an altered expression of circRNAs by cholesterol (Chol) in Neuro-2A, BV-2, and C8-D1a cells compared to each Ctr. The cropped band used for analysis is in **Supplementary Figures S4–S9**. In **A**–**F**, the data are described as the mean ± standard error of the mean (SEM) (n = 3), and statistical significance was determined using an unpaired two-tailed *t*-test with Welch's correction; *: $p < 0.05$, **: $p < 0.01$, ***: $p < 0.001$.

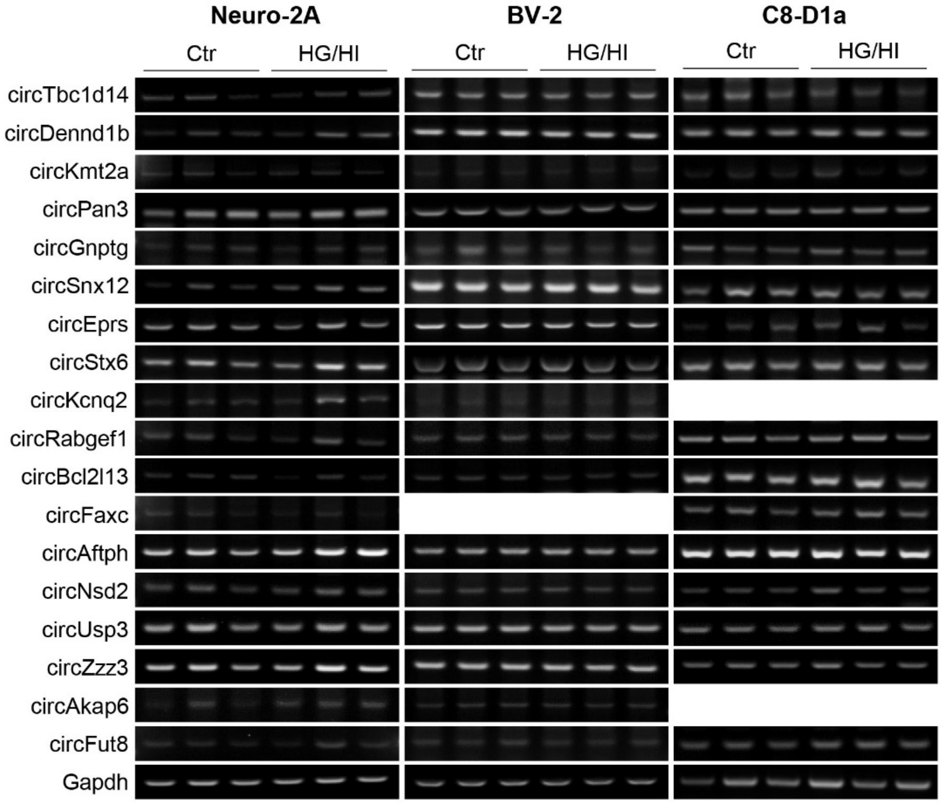

Supplementary Figure S3. Cropped bands showing the expression of circRNAs in the untreated control (Ctr) and high glucose and insulin concentration (HG/HI)-treated mouse neuroblastoma cells (Neuro-2A), mouse microglial cells (BV-2), and mouse astrocytes (C8-D1a). A space with no cropped band indicates that circRNAs were not expressed in this assay.

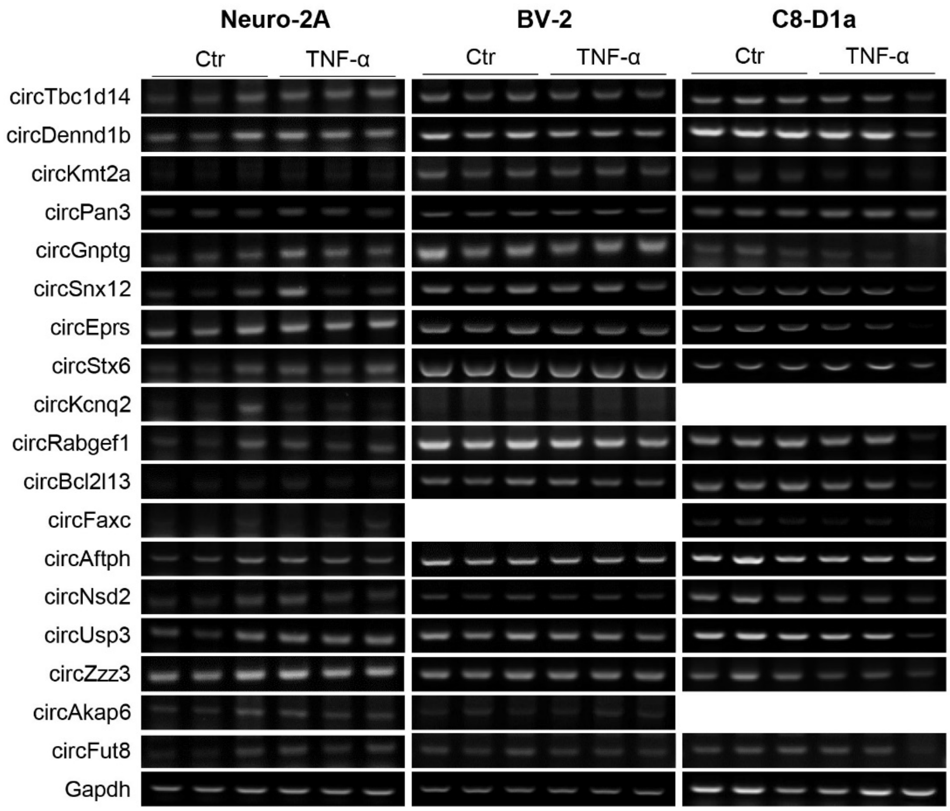

Supplementary Figure S4. Cropped bands showing the expression of circRNAs in the untreated control (Ctr) and tumor necrosis factor-alpha (TNF-α)-treated mouse neuroblastoma cells (Neuro-2A), mouse microglial cells (BV-2), and mouse astrocytes (C8-D1a). A space with no cropped band indicates that circRNAs were not expressed in this assay.

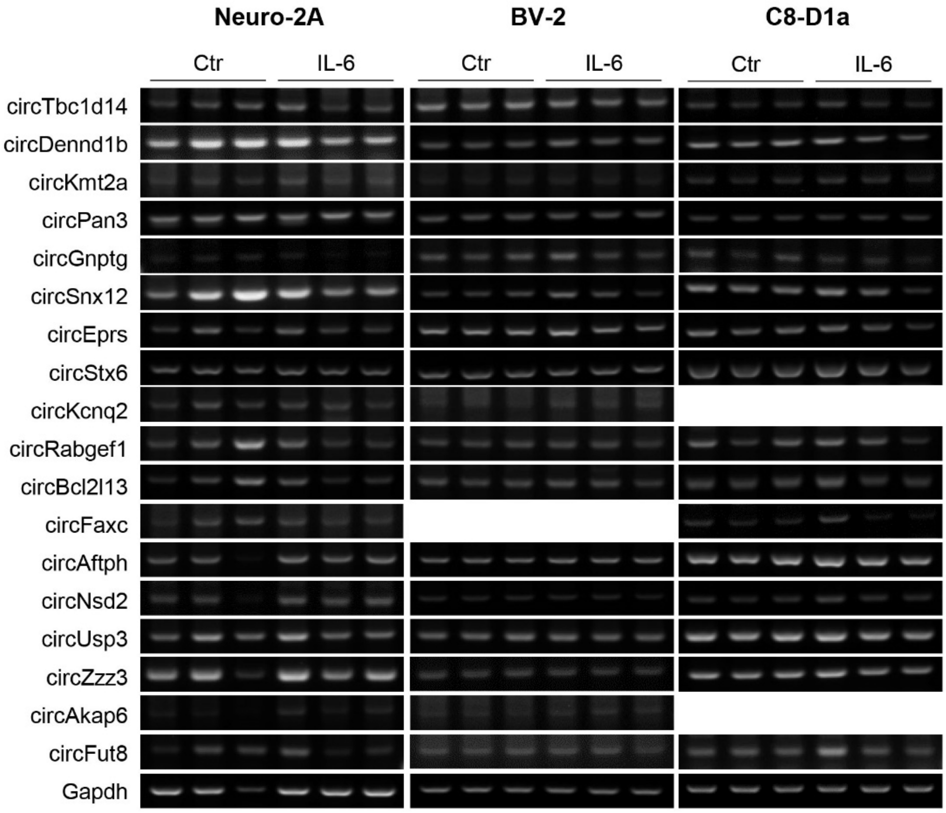

Supplementary Figure S5. Cropped bands showing the expression of circRNAs in the untreated control (Ctr) and interleukin-6 (IL-6)-treated mouse neuroblastoma cells (Neuro-2A), mouse microglial cells (BV-2), and mouse astrocytes (C8-D1a). A space with no cropped band indicates that circRNAs were not expressed in this assay.

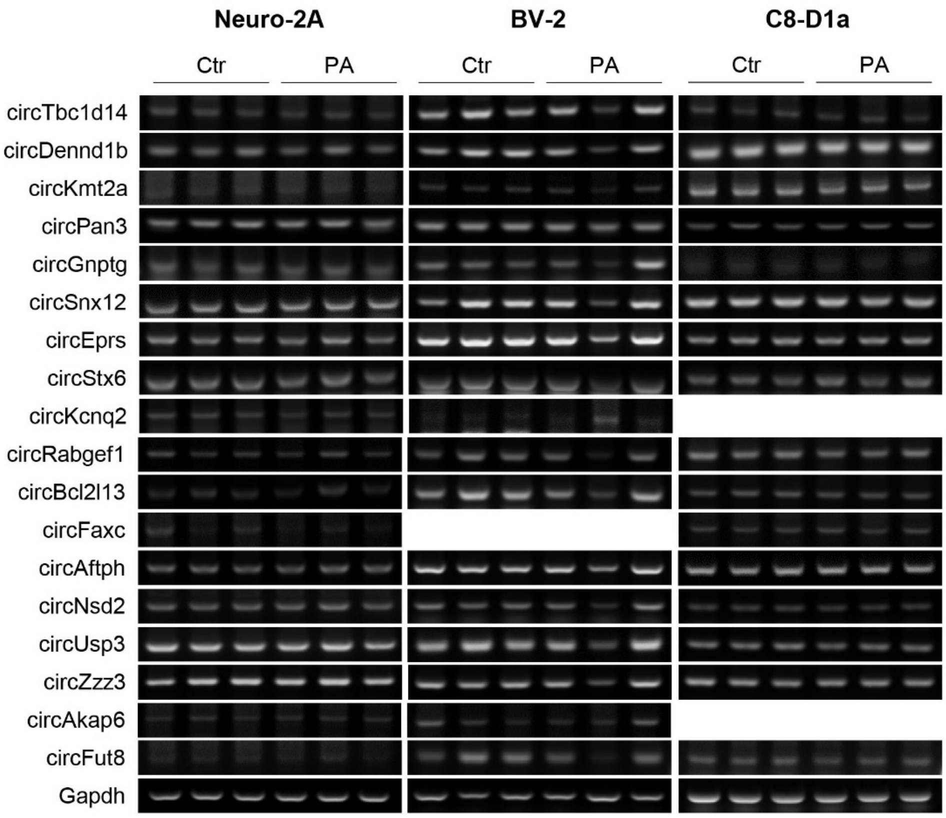

Supplementary Figure S6. Cropped bands showing the expression of circRNAs in the untreated control (Ctr) and BSA-conjugated palmitic acid (PA)-treated mouse neuroblastoma cells (Neuro-2A), mouse microglial cells (BV-2), and mouse astrocytes (C8-D1a). A space with no cropped band indicates that circRNAs were not expressed in this assay.

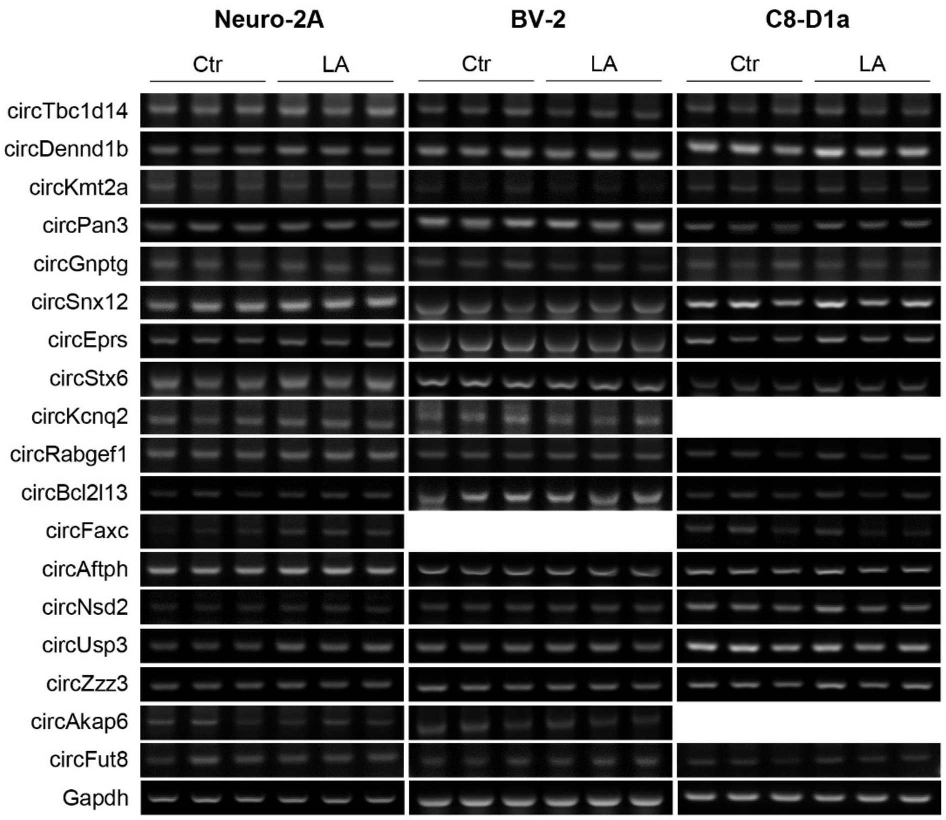

Supplementary Figure S7. Cropped bands showing the expression of circRNAs in the untreated control (Ctr) and BSA-conjugated linoleic acid (LA)-treated mouse neuroblastoma cells (Neuro-2A), mouse microglial cells (BV-2), and mouse astrocytes (C8-D1a). A space with no cropped band indicates that circRNAs were not expressed in this assay.

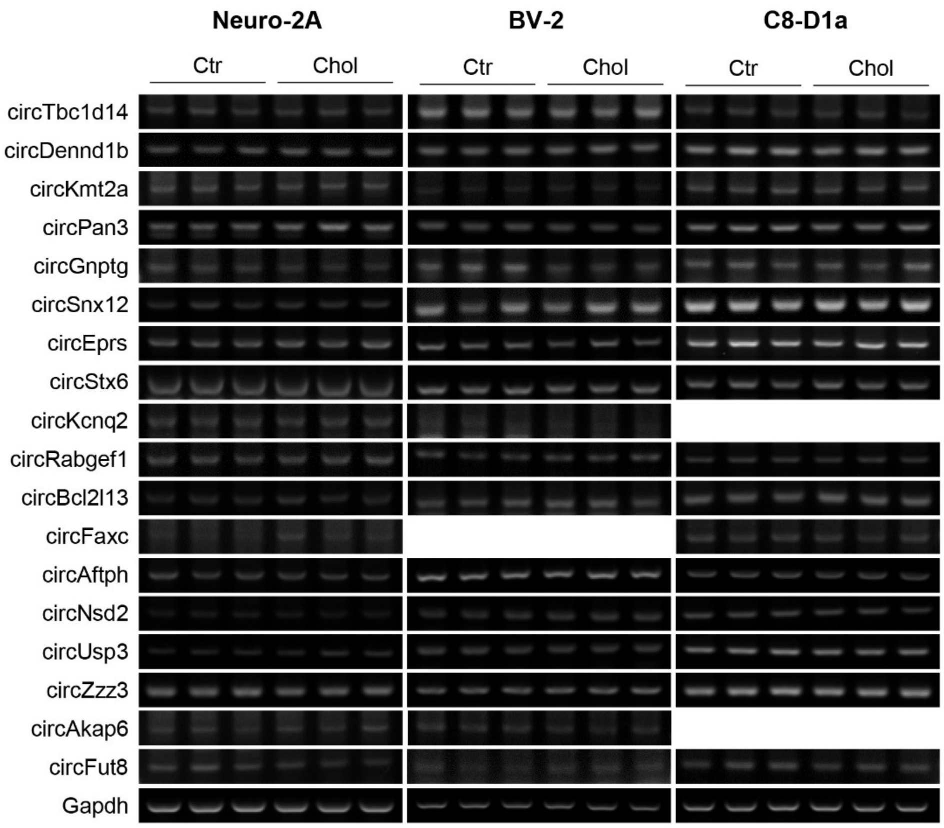

Supplementary Figure S8. Cropped bands showing the expression of circRNAs in the untreated control (Ctr) and cholesterol (Chol)-treated mouse neuroblastoma cells (Neuro-2A), mouse microglial cells (BV-2), and mouse astrocytes (C8-D1a). A space with no cropped band indicates that circRNAs were not expressed in this assay.

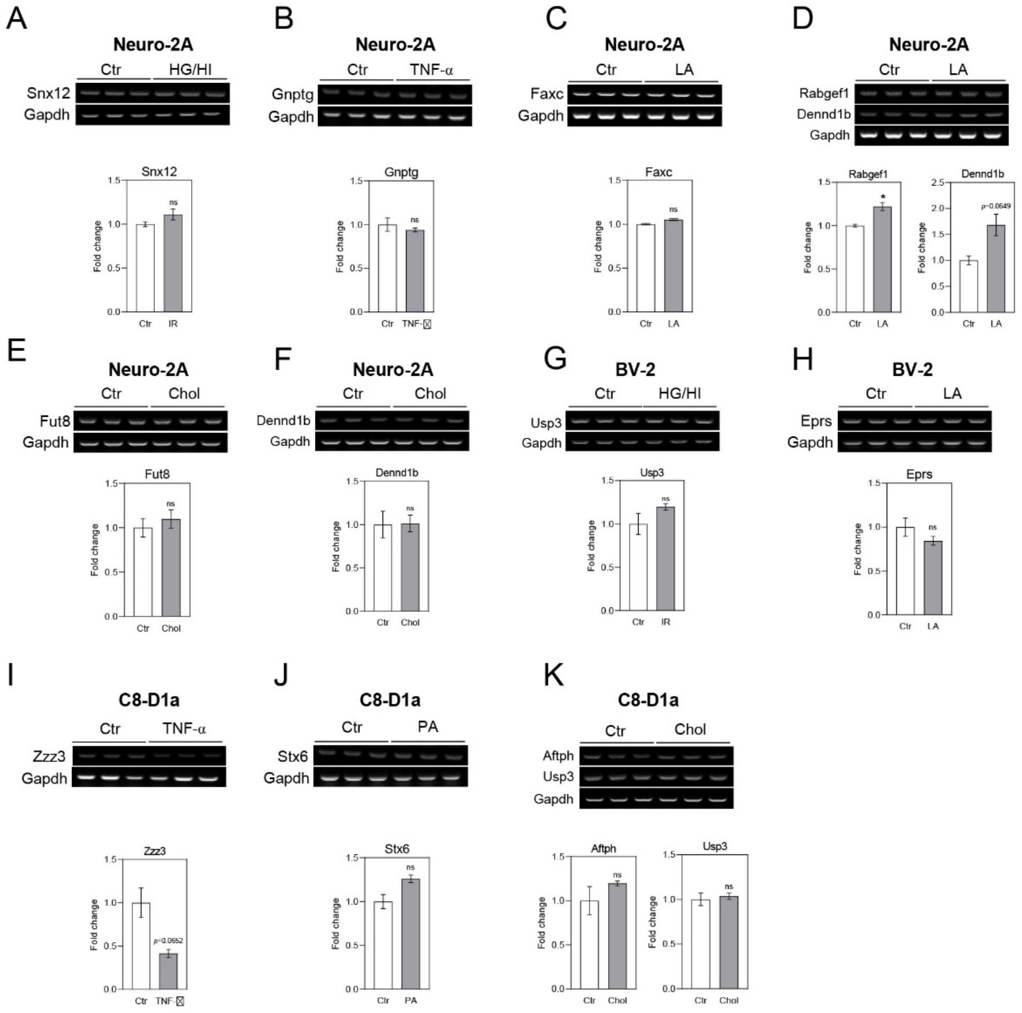

Supplementary Figure S9. The host gene expression corresponding to circRNAs of interest in three mouse-brain cell lines under obesity-like conditions. **A.** Cropped bands and a histogram show the expression of the host gene corresponding to circSnx12 in the untreated control (Ctr) and high glucose and insulin concentration (HG/HI)-treated mouse neuroblastoma cells (Neuro-2A). **B.** Cropped bands and a histogram show the expression of the host gene corresponding to circGnptg in Ctr and tumor necrosis factor-alpha (TNF-α)-treated Neuro-2A cells. **C.** Cropped bands and a histogram show the expression of the host gene corresponding

to circFaxc in Ctr and BSA-conjugated linoleic acid (LA)-treated Neuro-2A cells. **D.** Cropped bands and histograms show the expression of host genes corresponding to circRabgef1 and circDennd1b in Ctr and LA-treated Neuro-2A cells. **E.** Cropped bands and a histogram show the expression of the host gene corresponding to circFut8 in Ctr and cholesterol (Chol)-treated Neuro-2a cells. **F.** Cropped bands and a histogram show the expression of the host gene corresponding to circDennd1b in Ctr and Chol-treated Neuro-2A cells. **G.** Cropped bands and a histogram show the expression of the host gene corresponding to circUsp3 in Ctr and HG/HI-treated mouse microglial cells (BV-2). **H.** Cropped bands and a histogram show the expression of the host gene corresponding to circEprs in Ctr and LA-treated BV-2 cells. **I.** Cropped bands and histograms show the expression of host genes corresponding to circZzz3 in Ctr and TNF-α-treated mouse astrocytes (C8-D1a). **J.** Cropped bands and a histogram show the expression of the host gene corresponding to circStx6 in Ctr and BSA-conjugated palmitic acid (PA)-treated C8-D1a cells. **K.** Cropped bands and histograms show the expression of host genes corresponding to circAftph and circUsp3 in Ctr and Chol-treated C8-D1a cells. In **A**–**K**, the data are presented as the mean ± standard error of the mean (SEM) (n = 3). Statistical significance was determined using an unpaired two-tailed *t*-test with Welch's correction; ns: $p>0.05$ and *: $p<0.05$.

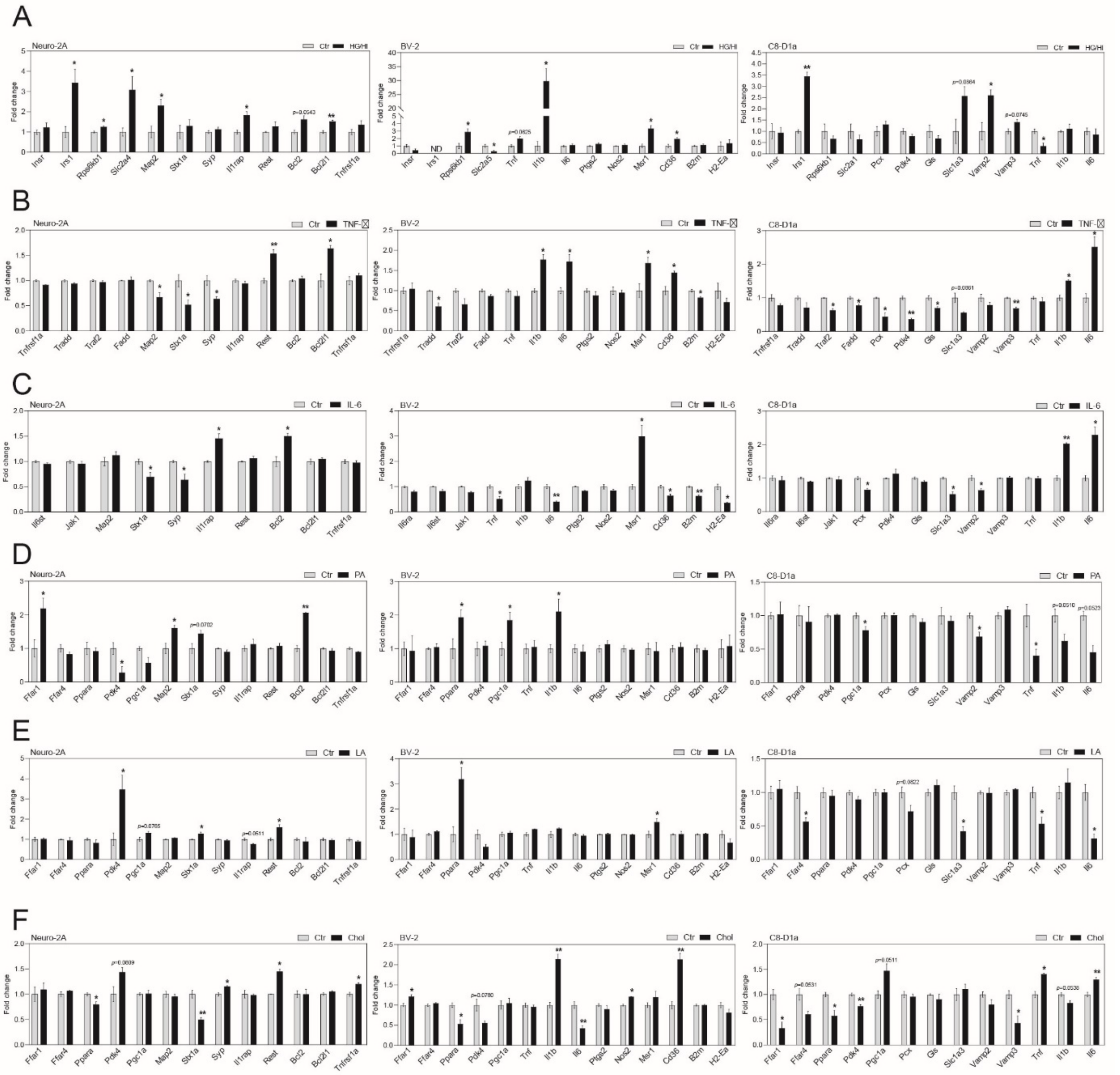

Supplementary Figure S10. Functional gene expression in obesity-like condition models for cell model verification. **A.** Histograms show altered gene expression by high glucose and insulin concentration (HG/HI) in mouse neuroblastoma cells (Neuro-2A), mouse microglial cells (BV-2), and mouse astrocytes (C8-D1a) compared to each untreated control (Ctr). **B.** Histograms show altered gene expression by tumor necrosis factor-alpha (TNF-α) in Neuro-2A, BV-2, and C8-D1a cells compared to each Ctr. **C.** Histograms show altered gene expression by interleukin-6 (IL-6) in Neuro-2A, BV-2, and C8-D1a cells compared to each Ctr. **D.**

Histograms show altered gene expression by BSA-conjugated palmitic acid (PA) in Neuro-2A, BV-2, and C8-D1a cells compared to each Ctr. **E.** Histograms show altered gene expression by BSA-conjugated linoleic acid (LA) in Neuro-2A, BV-2, and C8-D1a cells compared to each Ctr. **F.** Histograms show altered gene expression by cholesterol (Chol) in Neuro-2A, BV-2, and C8-D1a cells compared to each Ctr. In **A**–**F**, the data are presented as the mean ± standard error of the mean (SEM) (n = 3), and statistical significance was determined using an unpaired two-tailed *t*-test with Welch's correction; ns: $p > 0.05$, *: $p < 0.05$, **: $p < 0.01$, ***: $p < 0.001$.

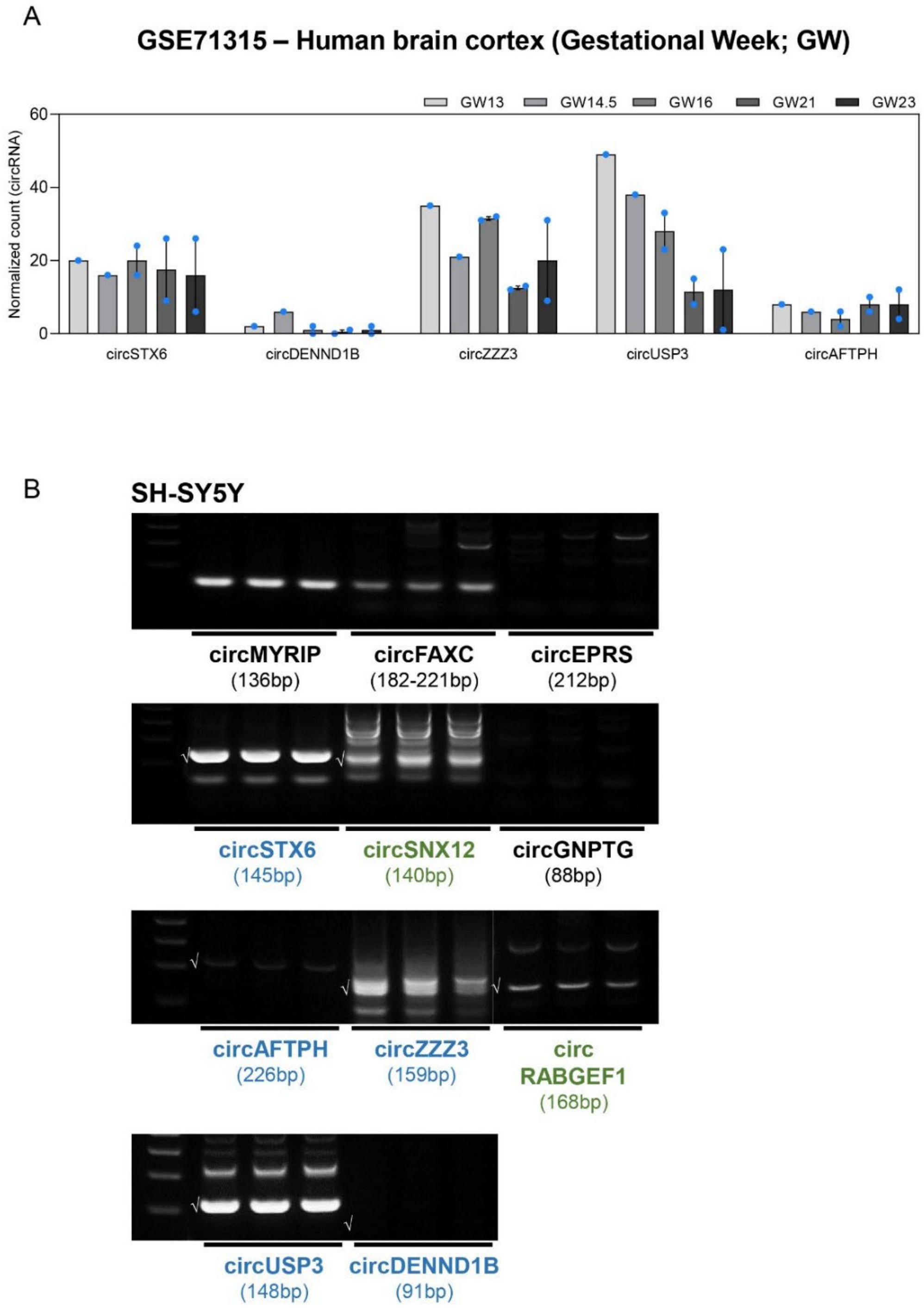

Supplementary Figure S11. CircRNA conservation in humans. **A.** A histogram displaying the normalized count of conserved circRNAs obtained from high throughput sequencing data of the gestational human brain cortex in the publicly available GEO dataset (GSE71315). GW indicates gestational week. The data are expressed as either a normalized count (n = 1; GW13

and GW14.5) or mean ± standard error of the mean (SEM) (n = 2; GW16, GW21, and GW23). The normalized count value of each circRNA is expressed as a blue dot in the histogram. **B.** Blots showing circRNA expression in human neuroblastoma cells (SH-SY5Y). The PCR product of each circRNA observed at the expected size is marked with a white square root on the blot. The blue and green texts indicate circRNAs observed in **A.** and circRNAs observed only in SH-SY5Y cells, respectively. The black text indicates circRNAs not expressed in the GEO dataset and SH-SY5Y cells.

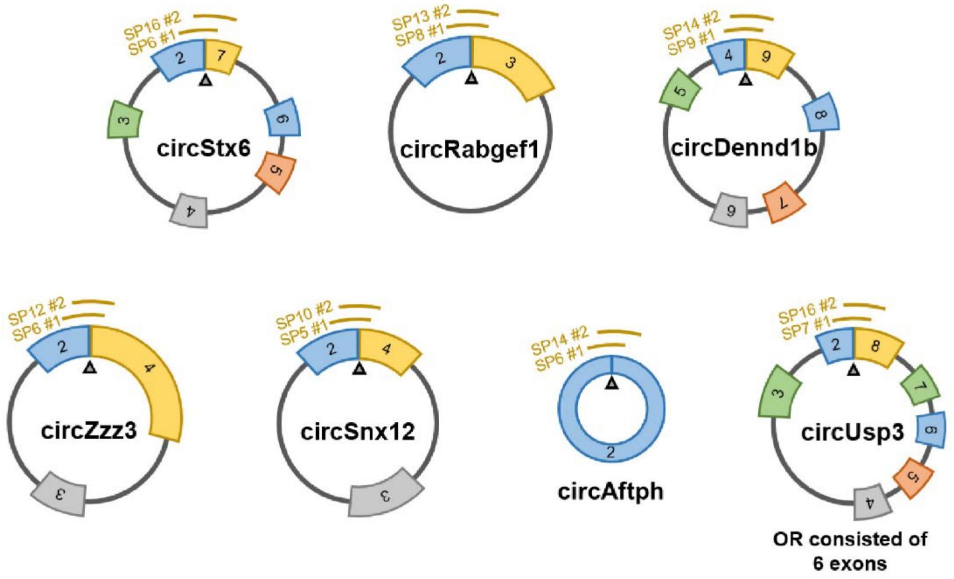

Supplementary Figure S12. Illustration showing the structures and siRNA-binding positions near the back-splicing junction site of circRNAs. The solid line and box of each circRNA model indicate the introns and exons, respectively. The number in the exon box represents the exon number designated in the Genome browser database. The silver triangle in each circRNA model indicates the back-splicing junction site. The gold curve near the back-splicing junction site of each circRNA indicates a siRNA-binding position. The siRNA was designed from 40 nucleotide sequences on the left (-20) and right (+20) sides of the back-splicing junction site (0). The starting position (SP) number 1 was counted from the -20 nucleotide on the left of the back-splicing junction site. The two custom siRNAs are denoted by #1 and #2, respectively.

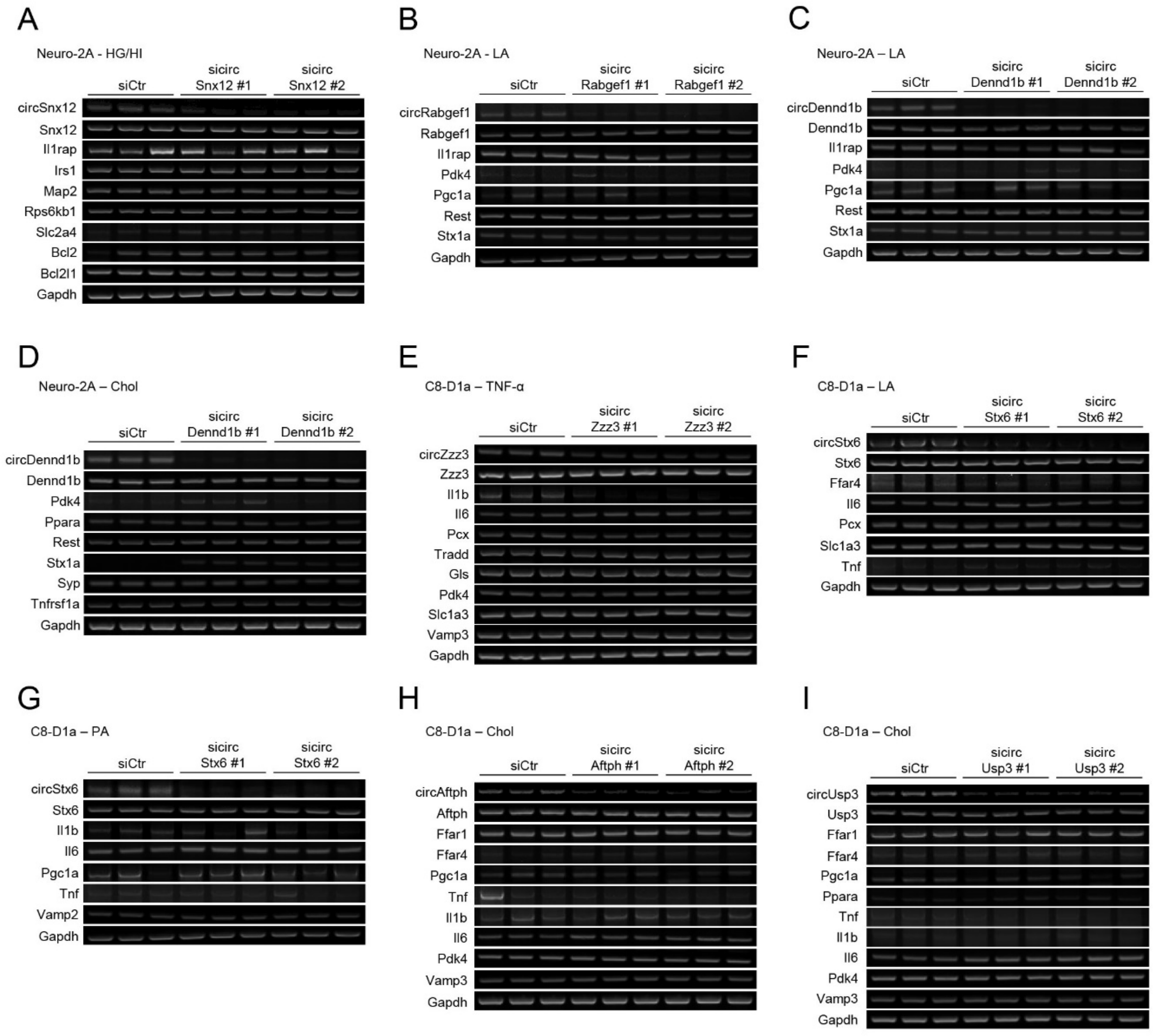

Supplementary Figure S13. Cropped bands showing the expression of circRNAs and genes in circRNA-depleted obesity-like condition models. **A.** Blots showing the expression of circSnx12 and genes in negative control siRNA (siCtr) and circSnx12 siRNAs (sicircSnx12)-transfected mouse neuroblastoma cells (Neuro-2A) under high glucose and insulin concentration (HG/HI). **B.** Blots showing the expression of circRabgef1 and genes in siCtr and circRabgef1 siRNAs (sicircRabgef1)-transfected Neuro-2A cells under BSA-conjugated linoleic acid (LA). **C.** Blots showing the expression of circDennd1b and genes in siCtr and circDennd1b siRNAs (sicircDennd1b)-transfected Neuro-2A cells under LA. **D.** Blots showing

the expression of circDennd1b and genes in siCtr and circDennd1b siRNAs (sicircDennd1b)-transfected Neuro-2A cells under cholesterol (Chol). **E.** Blots showing the expression of circZzz3 and genes in siCtr and circZzz3 siRNAs (sicircZzz3)-transfected C8-D1a cells under tumor necrosis factor-alpha (TNF-α). **F.** Blots showing the expression of circStx6 and genes in siCtr and circStx6 siRNAs (sicircStx6)-transfected C8-D1a cells under LA. **G.** Blots showing the expression of circStx6 and genes in siCtr and circStx6 siRNAs (sicircStx6)-transfected C8-D1a cells under BSA-conjugated palmitic acid (PA). **H.** Blots showing the expression of circAftph and genes in siCtr and circAftph siRNAs (sicircAftph)-transfected C8-D1a cells under Chol. **I.** Blots showing the expression of circUsp3 and genes in siCtr and sicircUsp3-transfected C8-D1a cells under Chol. The two custom siRNAs are denoted by #1 and #2, respectively.
