# Supplementary figures and images for: Profiling and Cellular Analyses of Obesity-Related circRNAs in Neurons and Glia under Obesity-like In Vitro Conditions
Source: Int J Mol Sci. 2023 Mar 25;24(7):6235. doi: 10.3390/ijms24076235 (PMC10094513; doi:10.3390/ijms24076235)

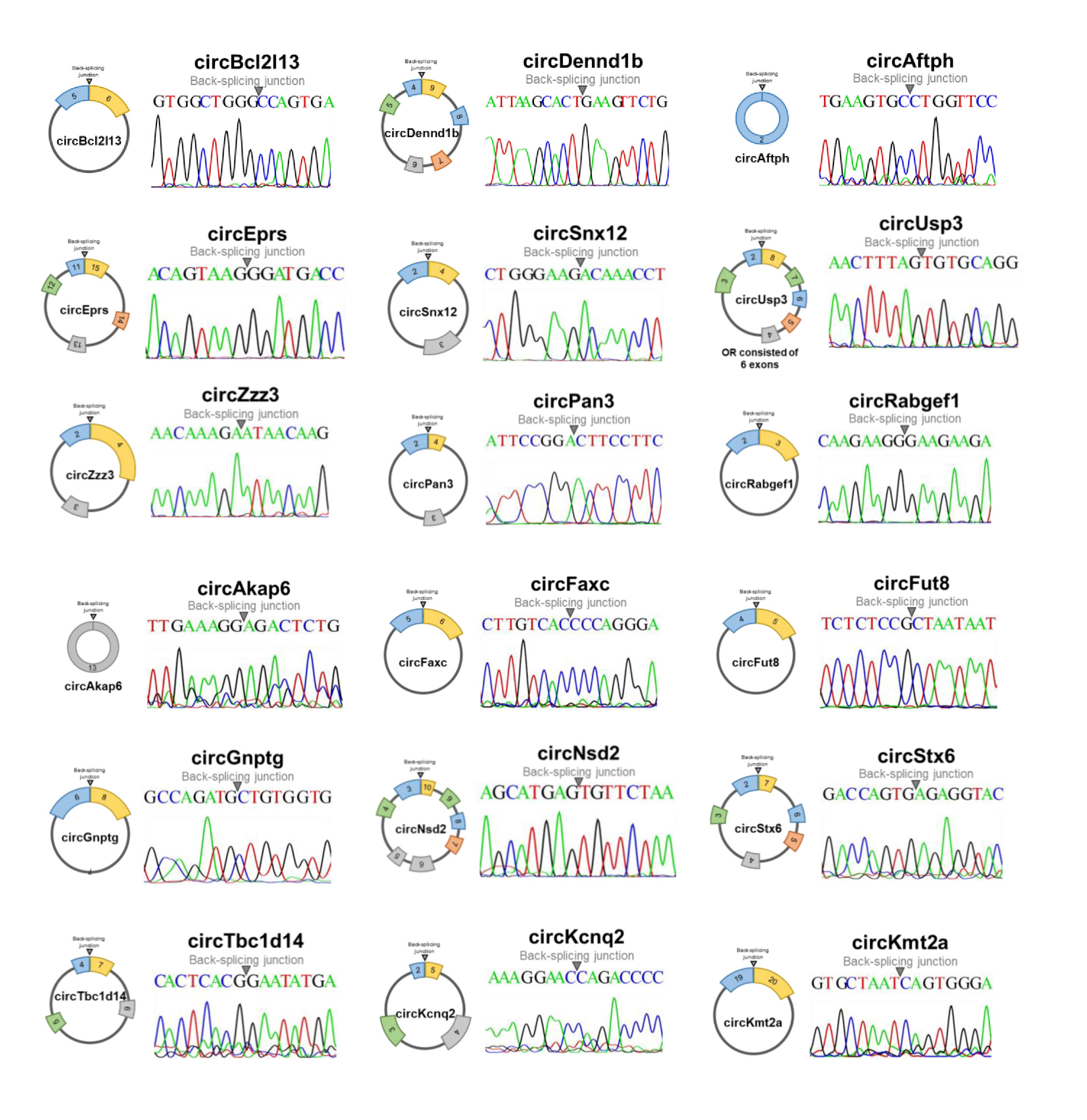

Supplement: Supplementary file 1 [file ijms-24-06235-s001.zip › supplementary files/Supplementary Figure 1.jpg]

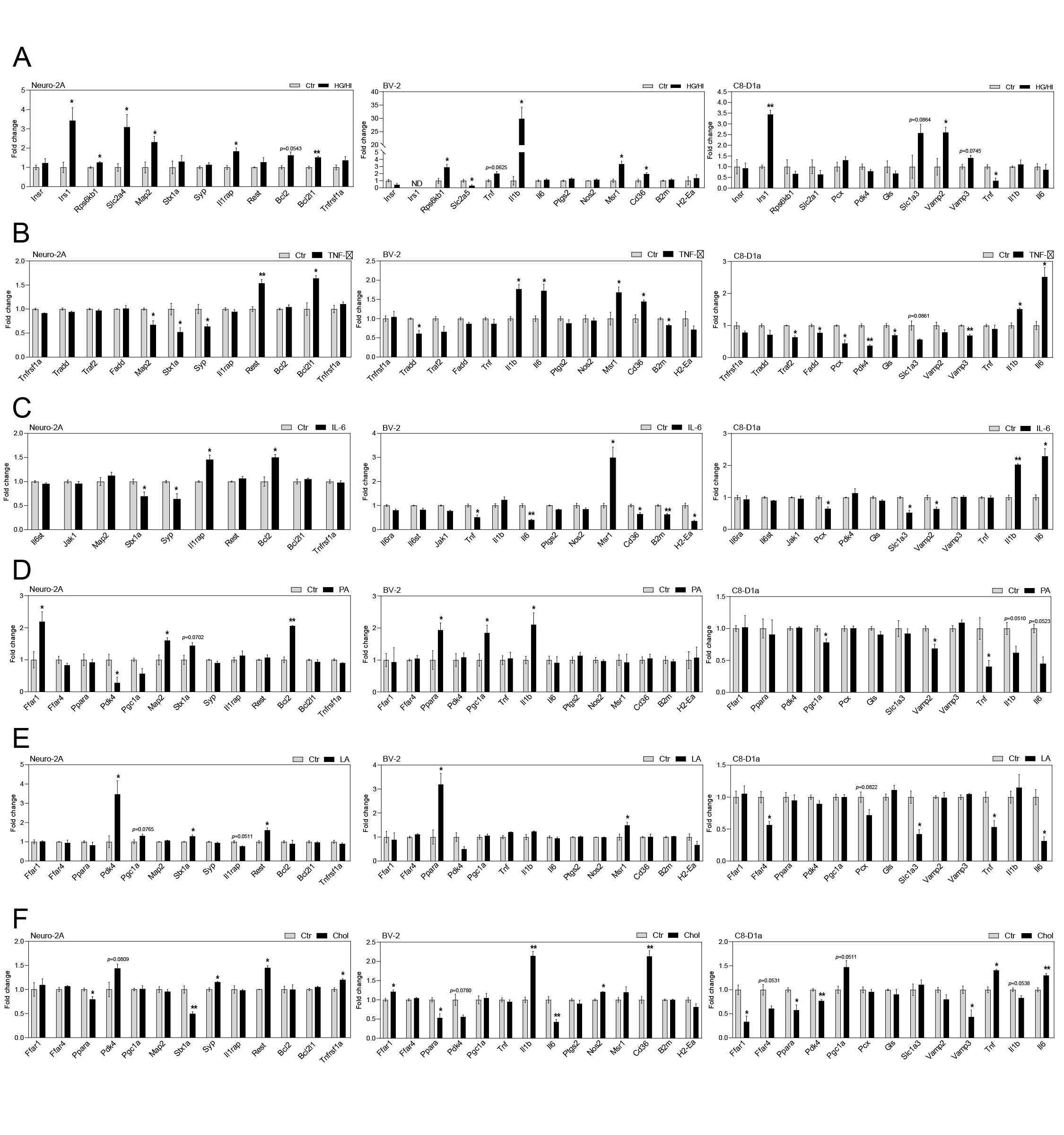

Supplement: Supplementary file 1 [file ijms-24-06235-s001.zip › supplementary files/Supplementary Figure 10.jpg]

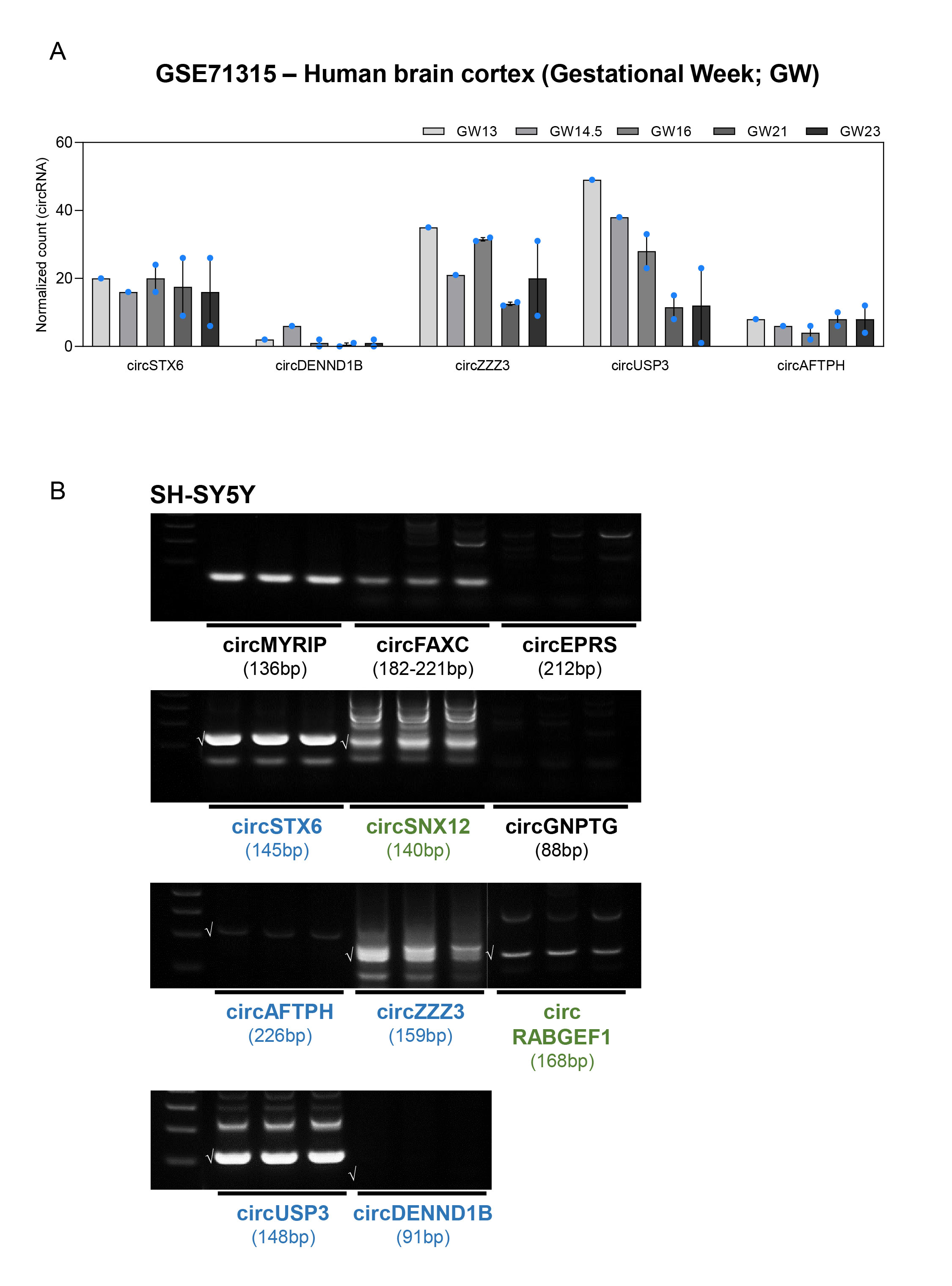

Supplement: Supplementary file 1 [file ijms-24-06235-s001.zip › supplementary files/Supplementary Figure 11.jpg]

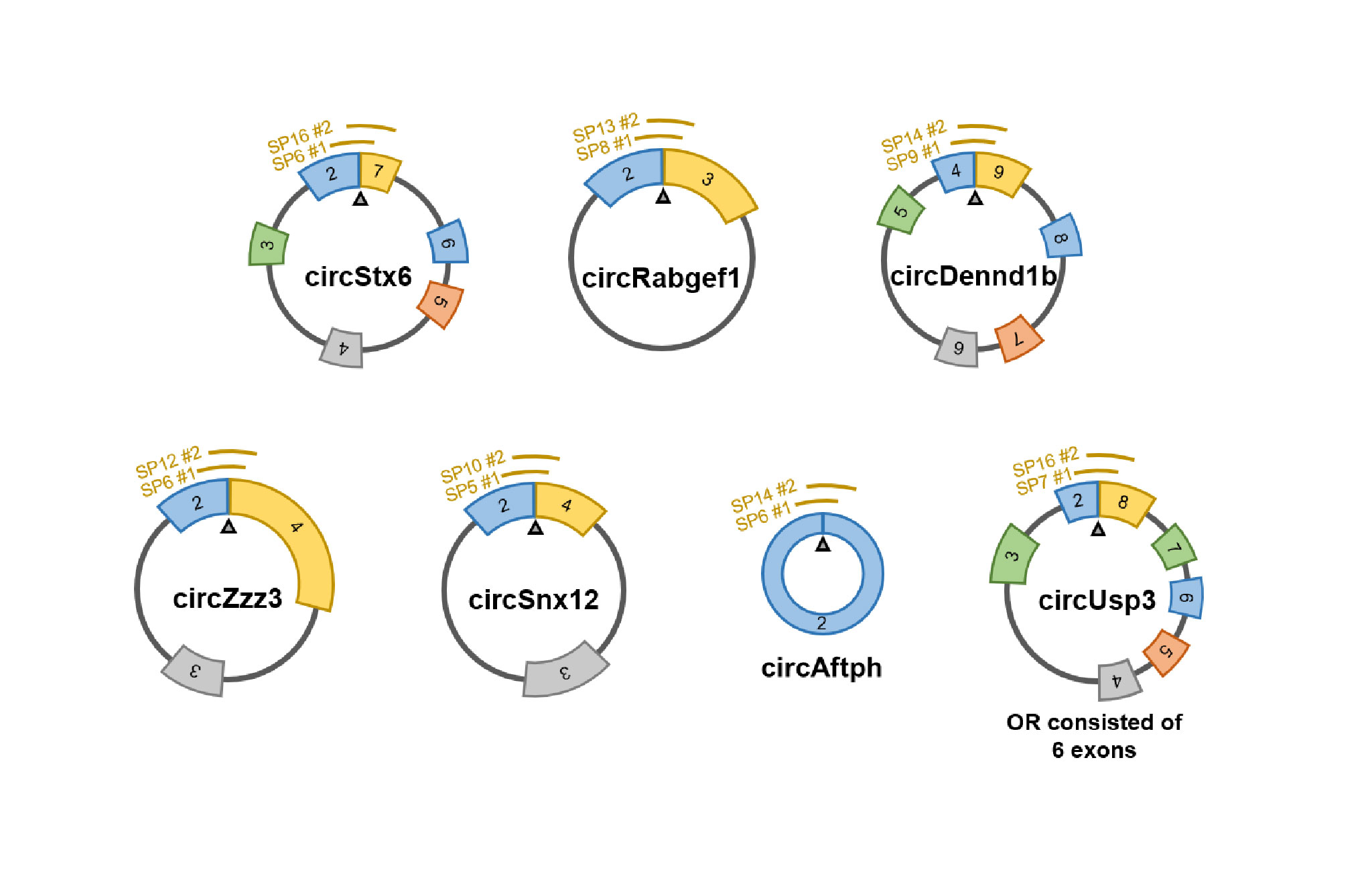

Supplement: Supplementary file 1 [file ijms-24-06235-s001.zip › supplementary files/Supplementary Figure 12.jpg]

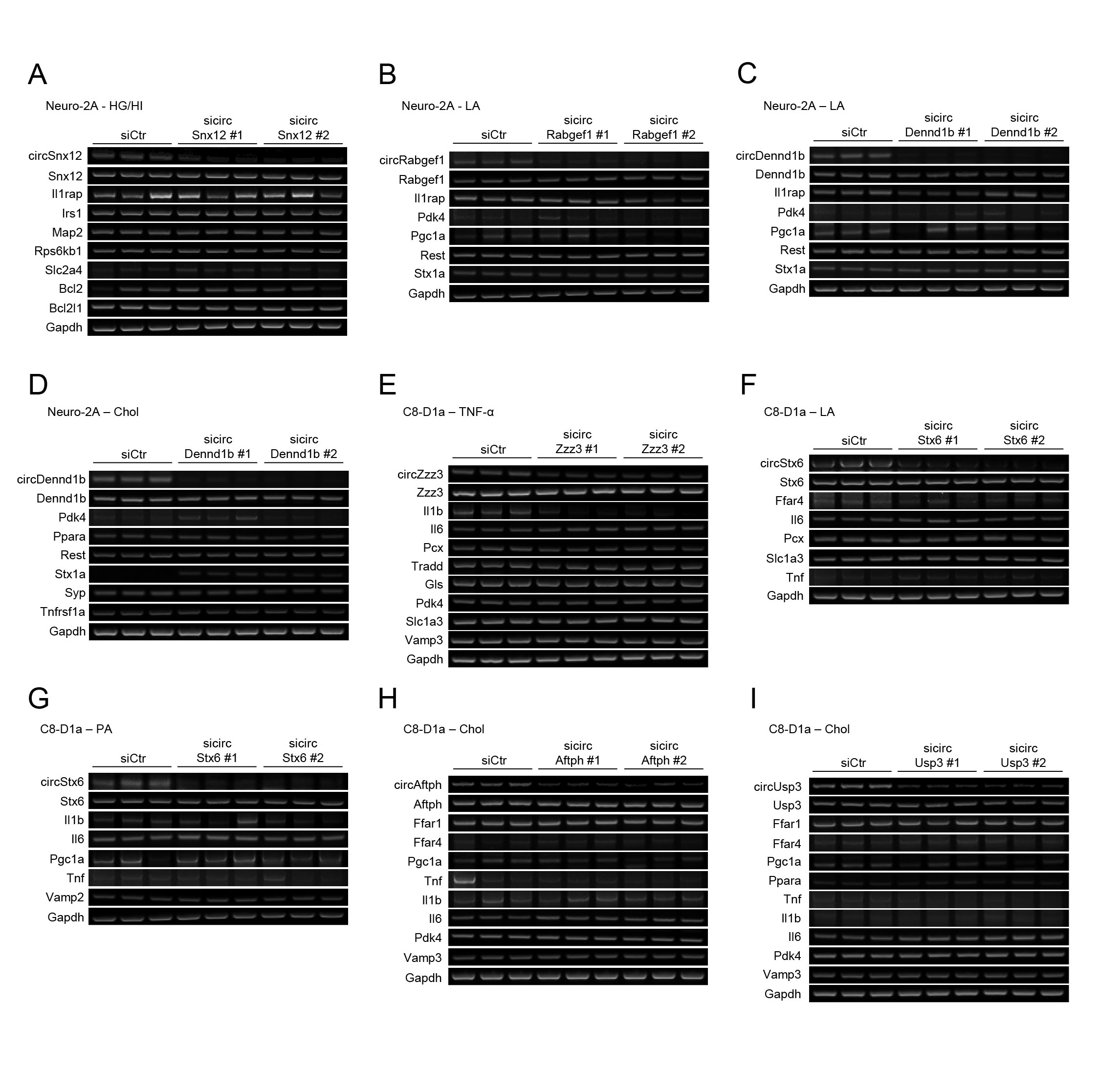

Supplement: Supplementary file 1 [file ijms-24-06235-s001.zip › supplementary files/Supplementary Figure 13.jpg]

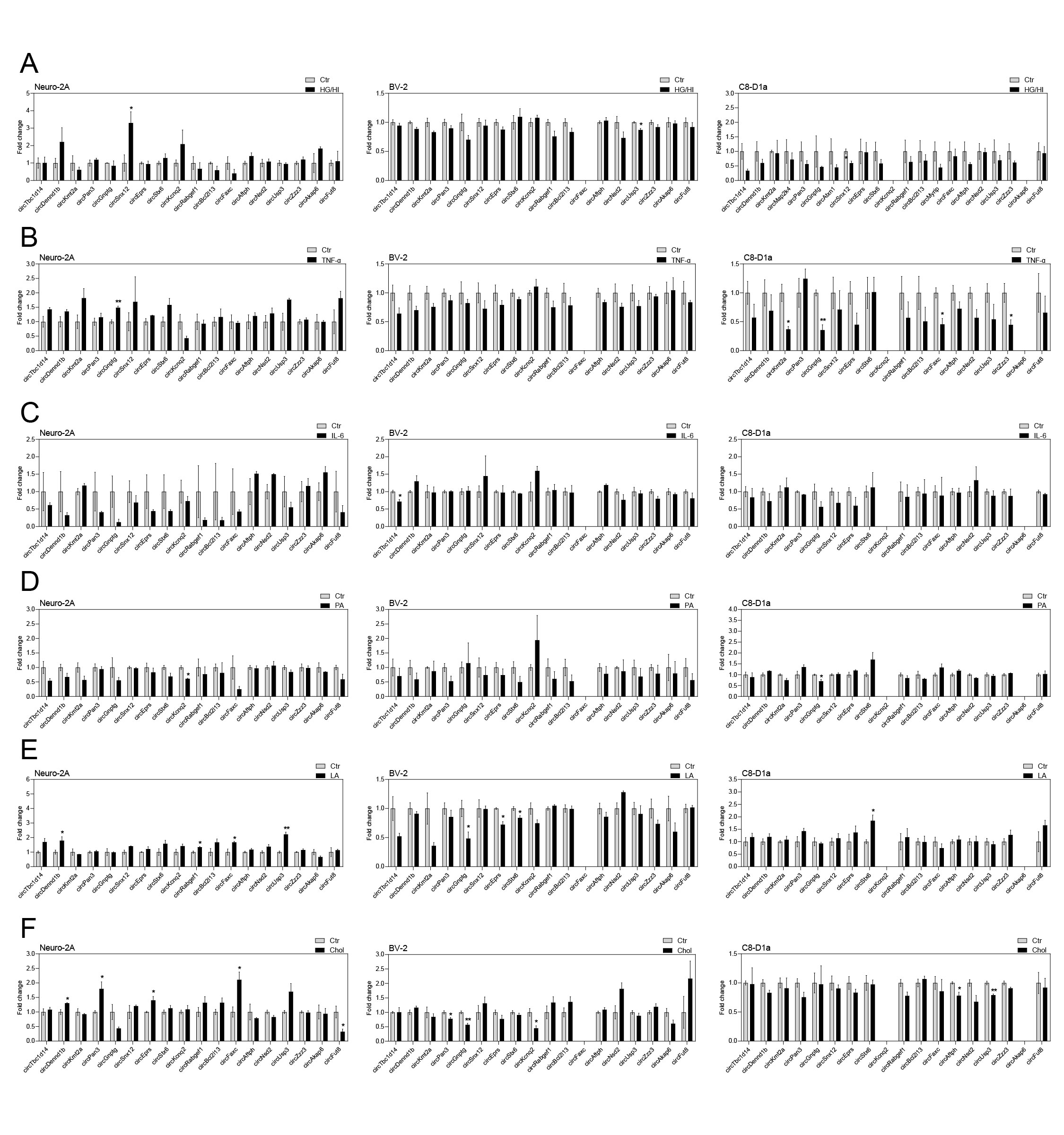

Supplement: Supplementary file 1 [file ijms-24-06235-s001.zip › supplementary files/Supplementary Figure 2.jpg]

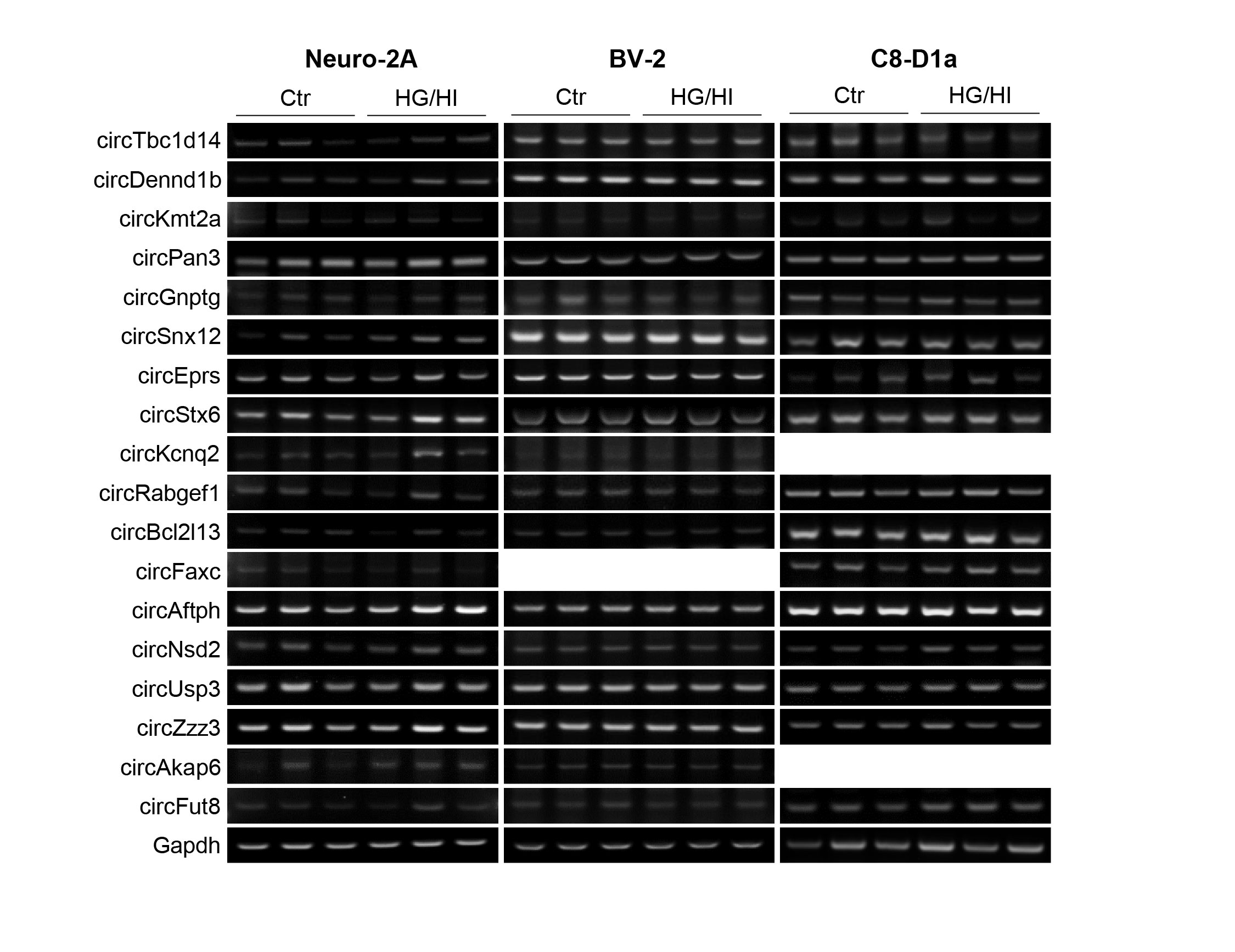

Supplement: Supplementary file 1 [file ijms-24-06235-s001.zip › supplementary files/Supplementary Figure 3.jpg]

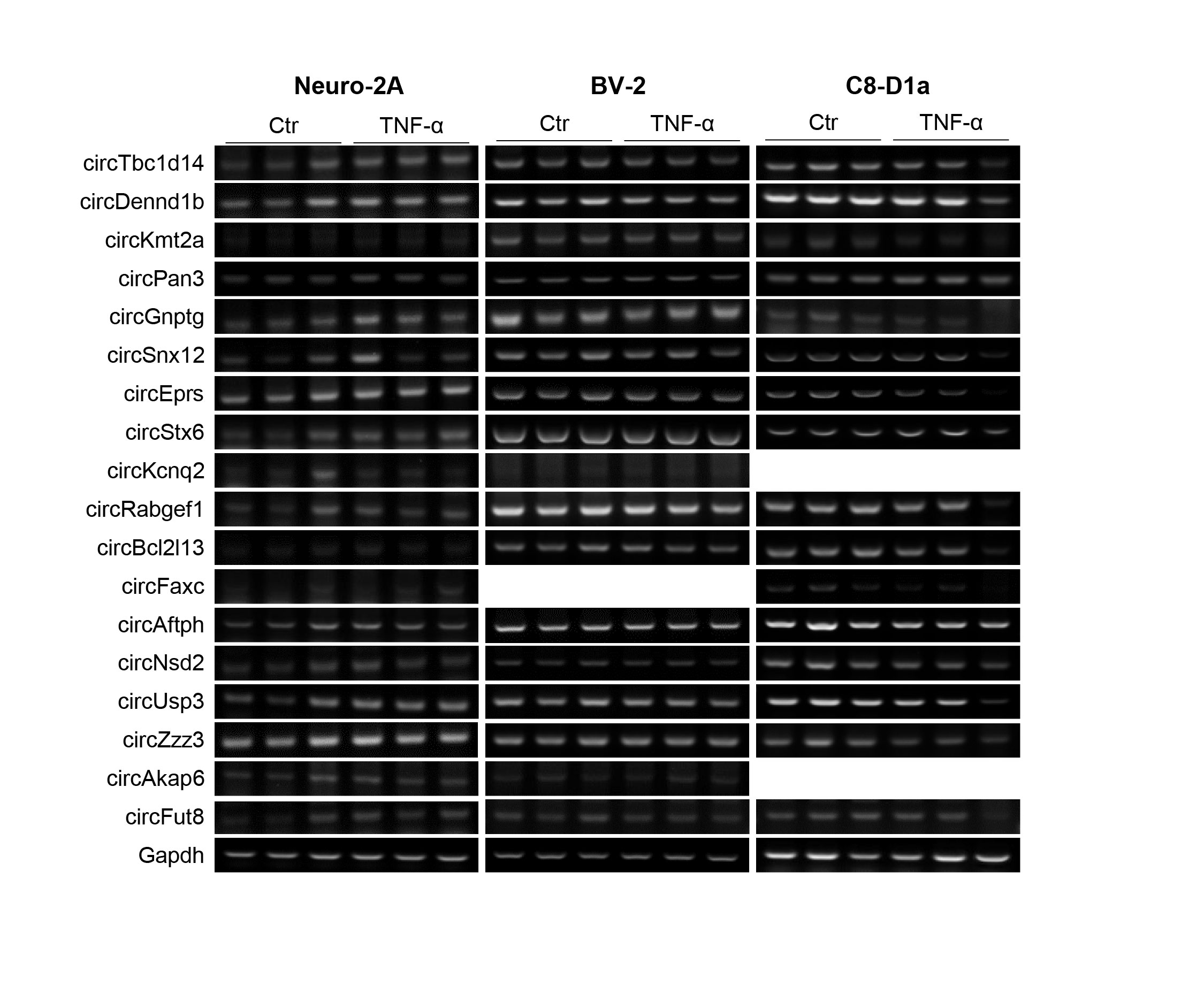

Supplement: Supplementary file 1 [file ijms-24-06235-s001.zip › supplementary files/Supplementary Figure 4.jpg]

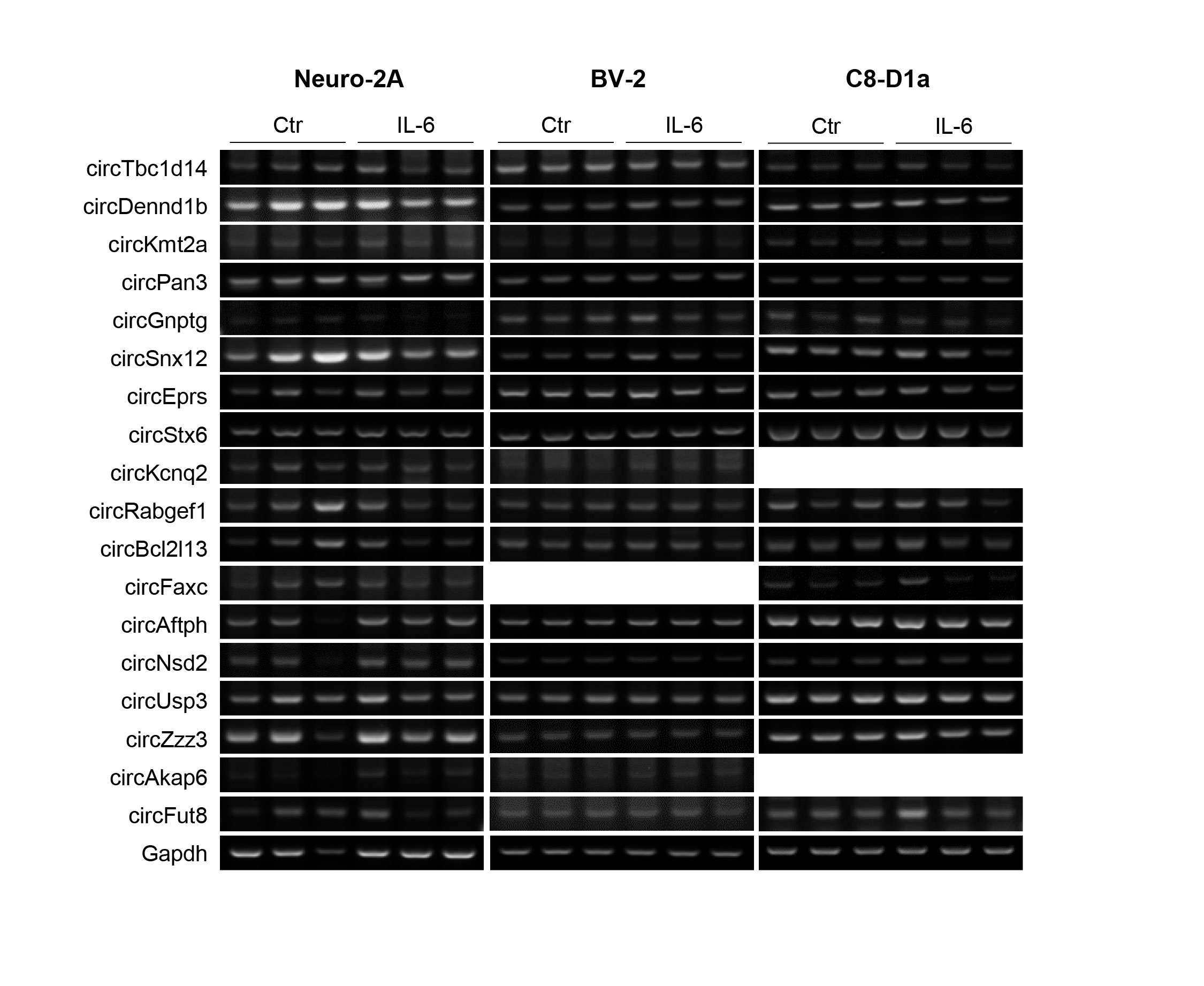

Supplement: Supplementary file 1 [file ijms-24-06235-s001.zip › supplementary files/Supplementary Figure 5.jpg]

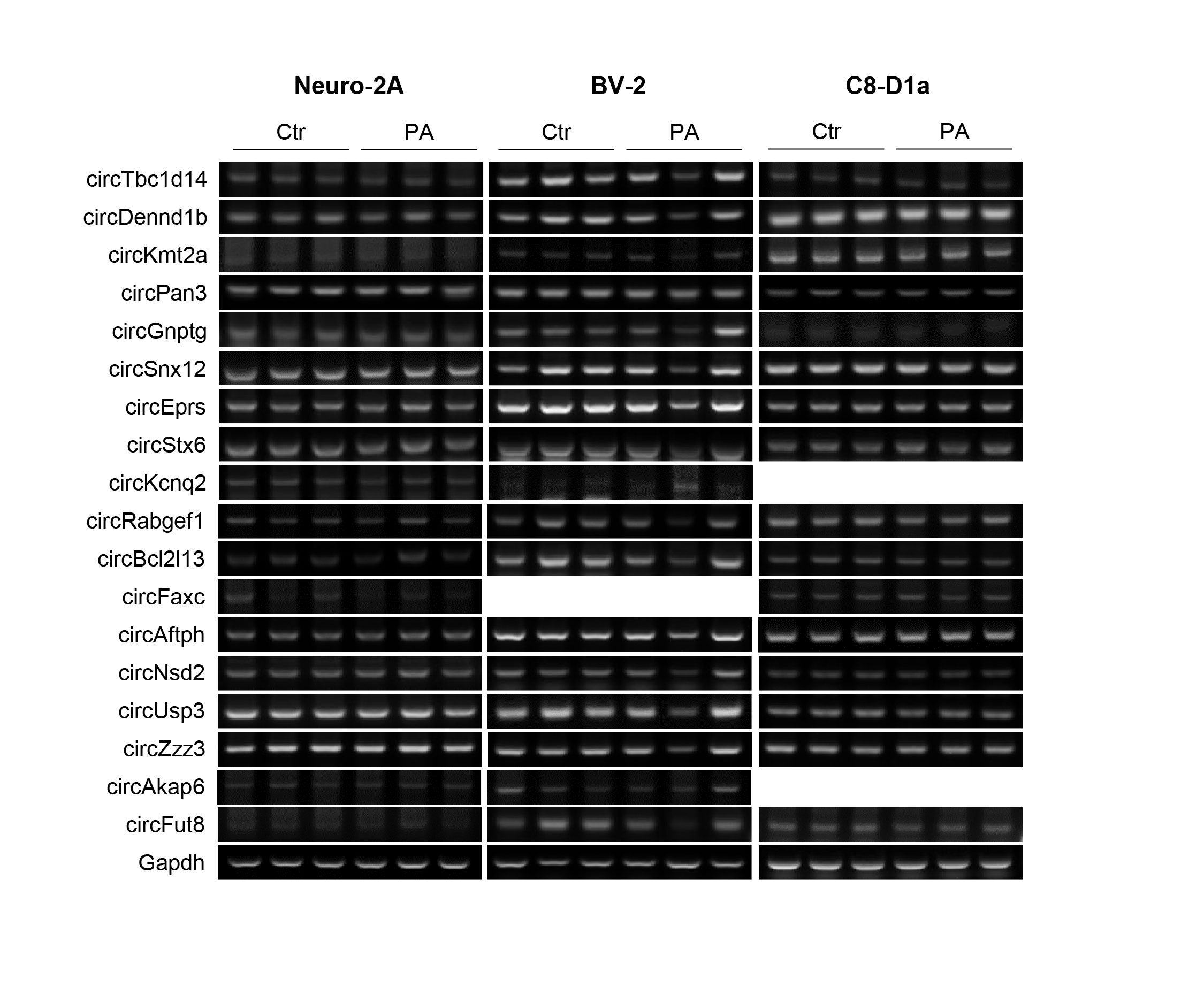

Supplement: Supplementary file 1 [file ijms-24-06235-s001.zip › supplementary files/Supplementary Figure 6.jpg]

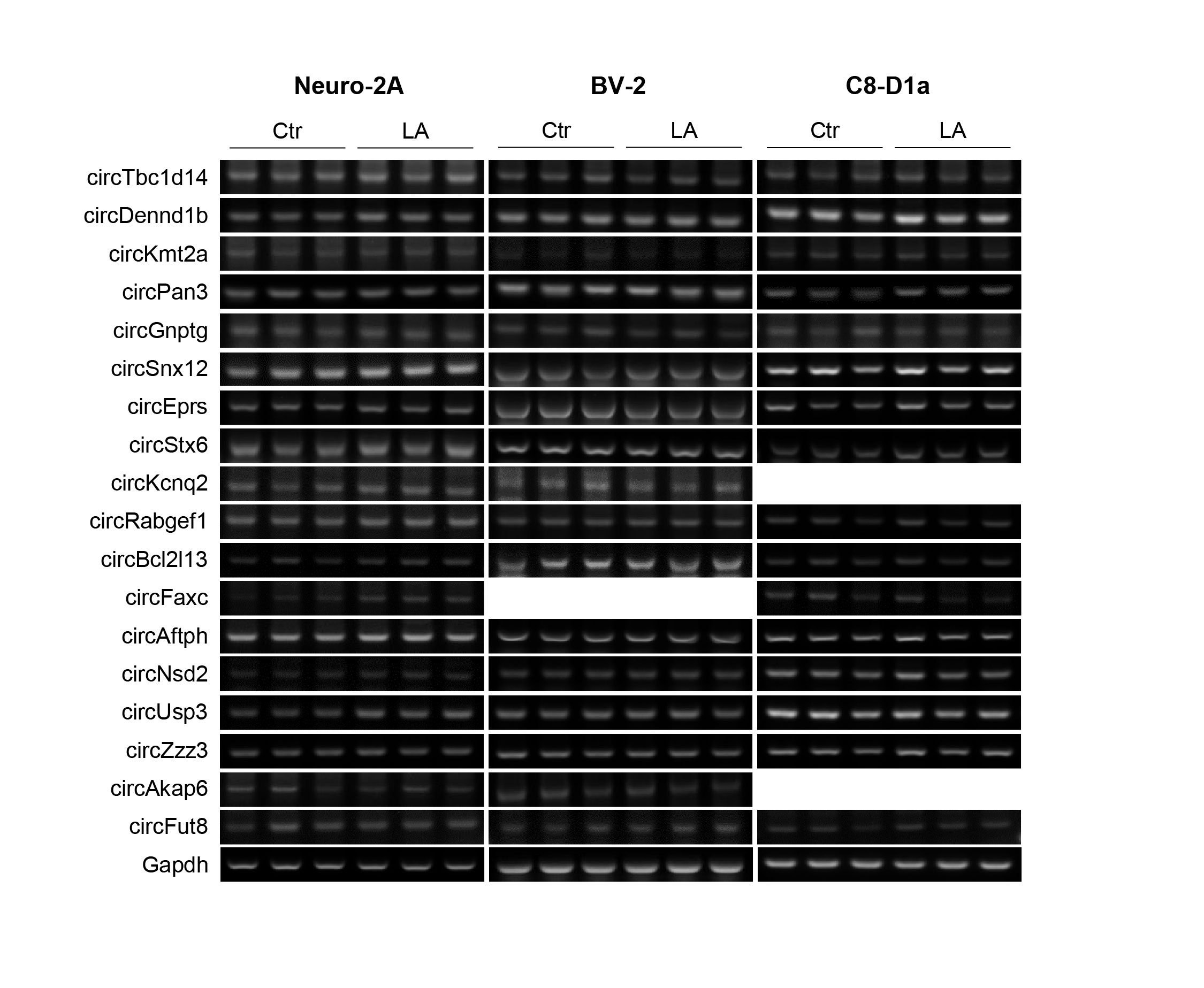

Supplement: Supplementary file 1 [file ijms-24-06235-s001.zip › supplementary files/Supplementary Figure 7.jpg]

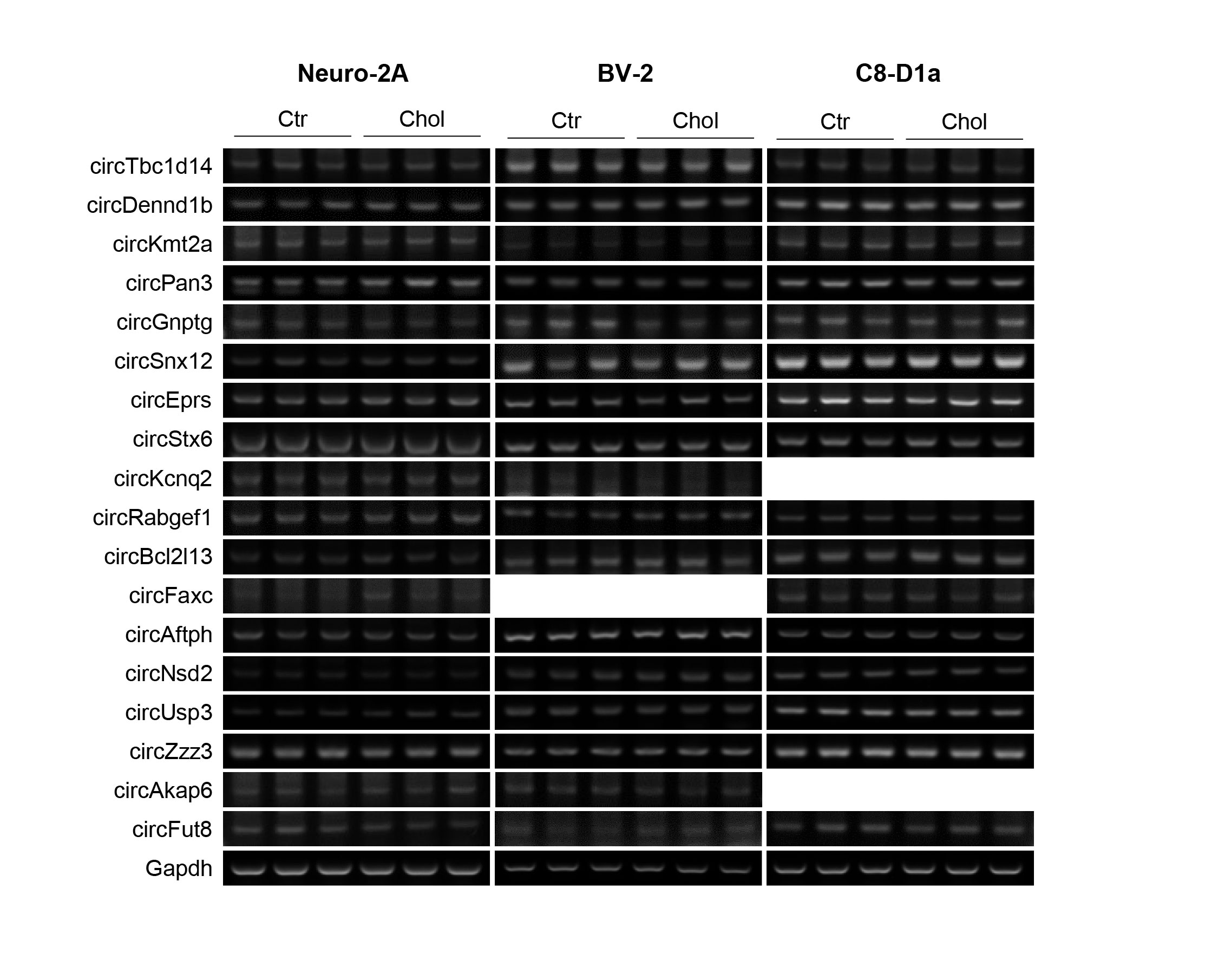

Supplement: Supplementary file 1 [file ijms-24-06235-s001.zip › supplementary files/Supplementary Figure 8.jpg]

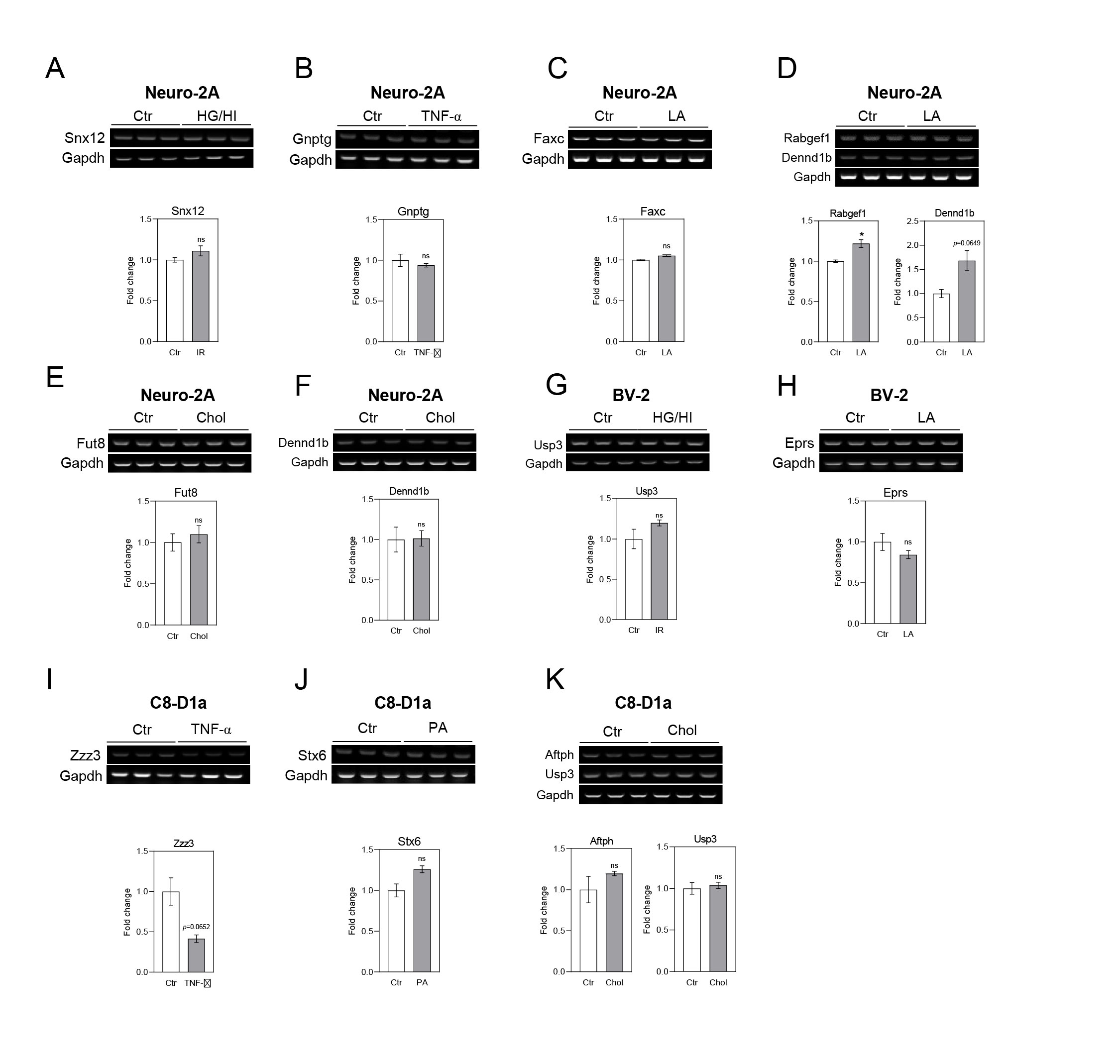

Supplement: Supplementary file 1 [file ijms-24-06235-s001.zip › supplementary files/Supplementary Figure 9.jpg]
